# AD-Like Neuropsychiatric Dysfunction in a Mice Model Induced by a Combination of High-Fat Diet and Intraperitoneal Injection of Streptozotocin

**DOI:** 10.1523/ENEURO.0310-24.2024

**Published:** 2024-12-10

**Authors:** Huaizhi Sun, Xinran Gao, Jiachun Niu, Pengquan Chen, Shuai He, Songlin Xu, Jinfang Ge

**Affiliations:** ^1^School of Pharmacy, Anhui Medical University, Hefei 230032, PR China; ^2^The Key Laboratory of Anti-inflammatory and Immune Medicine, Ministry of Education, Anhui Medical University, Hefei 230032, PR China; ^3^Anhui Provincial Laboratory of Inflammatory and Immune Disease, Anhui Institute of Innovative Drugs, Hefei 230032, PR China

**Keywords:** AD, cognitive function, microglia, neuroinflammation, TREM1/2

## Abstract

Increasing data suggest a crucial relationship between glycolipid metabolic disorder and neuropsychiatric injury. The aim of this study is to investigate the behavioral performance changes and neuropathological injuries in mice challenged with high-fat diet (HFD) and streptozotocin (STZ). The glucose metabolism indicators and behavioral performance were detected. The mRNA expression of IL-1β, IL-6, TNF-α, ocln, zo-1, and clnds and protein expression of APP, p-Tau, p-IRS1, p-AKT, p-ERK, and TREM1/2 were measured. The fluorescence intensities of MAP-2, NeuN, APP, p-Tau, GFAP, and IBA-1 were observed. The results showed that combination of HFD and STZ/I.P. could induce glucose metabolic turmoil and Alzheimer's disease (AD)-like neuropsychiatric dysfunction in mice, as indicated by the increased concentrations of fasting blood glucose and impaired learning and memory ability. Moreover, the model mice presented increased levels of APP, p-Tau, p-IRS1, TREM2, IL-1β, IL-6, TNF-α, ocln, zo-1, and clnds; decreased levels of p-AKT, p-ERK, and TREM1; and neuron damage and the hyperactivation of astrocytes and microglia in the hippocampus as compared with control mice. Only male mice were used in this study. Although AD and type 2 diabetes mellitus (T2DM) are distinct pathologies, our results suggested that combination of HFD and STZ/I.P., a widely used T2DM modeling method, could successfully induce AD-like behavioral impairments and neuropathological injuries in mice; the mechanism might be involved with neuroinflammation and its associated dysfunction of IRS1/AKT/ERK signaling pathway. Our findings further support the potential overlap between T2DM and AD pathophysiology, providing insight into the mechanisms underlying the comorbidity of these diseases.

## Significance Statement

Alzheimer's disease (AD) is a progressive neurodegenerative disorder whose prevalence is increasing with the rapidly growing global elderly population, but the accurate disease mechanisms and underlying therapeutic targets remain unclear. The aim of the present study is to investigate the behavioral performance changes and neuropathological injuries in mice challenged with the combination of a high-fat diet (HFD) and intraperitoneal injection of streptozotocin (STZ/I.P.). Our study suggested that combination of HFD and STZ/I.P. could successfully induce AD-like behavioral impairments and neuropathological injuries in mice, whose mechanism might be involved with neuroinflammation and its associated dysfunction of the IRS1/AKT/ERK signaling pathway. Our findings further support the potential overlap between T2DM and AD pathophysiology, providing insight into the mechanisms underlying the comorbidity of these diseases.

## Introduction

Alzheimer's disease (AD) is a progressive neurodegenerative disorder affecting elders globally with an increasing prevalence (Scheltens et al., 2016). According to the report from World Health Organization (WHO), approximately 5% of individuals aged 65 and older worldwide are affected by AD, and it has been estimated up to 115 million individuals worldwide living with AD by 2050 (Sengoku, 2020). However, only five treatment options are currently approved in the United States to address the cognitive symptoms of AD, with the most recent approval being memantine over a decade ago (Cummings et al., 2014). Regrettably, in the last 15 years, the majority of treatments under development have proven unsuccessful, with notable examples like aducanumab, which despite FDA approval, exhibited a striking 99.6% failure rate (Briggs et al., 2016). This highlights the critical necessity for advancements in comprehending disease mechanisms and pinpointing therapeutic targets.

Although the mechanism underlying the onset and progression of AD is not fully clear, mutations of amyloid precursor protein that result in abnormal production of Aβ peptides (Ghiso et al., 2004), and the intracellular formation of neurofibrillary tangles (NFTs) in the brain (J. Z. Wang and Liu, 2008), have been suggested to play a dominant role in the pathogenesis of AD (Maldonado-Díaz et al., 2024). Besides, recent studies have suggested a potential link between AD and brain insulin resistance (IR; Craft et al., 1998; Arnold et al., 2018; Kshirsagar et al., 2021). Moreover, increasing data suggested a close relation between metabolic disorders, especially diabetes mellitus, and the development of AD (Chakrabarti et al., 2015; Ashton et al., 2024). It has been reported that patients with type 2 diabetes mellitus (T2DM) have more than twice the risk of developing late-onset AD compared with nondiabetic individuals (Zhou et al., 2014) and that 80% of AD patients also exhibit symptoms of T2DM or abnormal glucose and insulin levels (S. Gao et al., 2019). It has been reported that the decreased insulin levels and impaired insulin signaling in the brain of AD patients could lead to the dysfunctions of glucose metabolism and synaptic plasticity, and the formation of NFTs and amyloid plaques, which are the critical processes involved in AD progression (Rad et al., 2018; Chen et al., 2023). Interestingly, accumulated Aβ and hyperphosphorylated tau protein have also been observed in the pancreatic tissue of T2DM patients and brain tissue of T2DM mice (Baglietto-Vargas et al., 2016; Marciniak et al., 2017). Therefore, understanding the intricate interplay between Aβ plaques, NFTs, and IR in the AD process may provide valuable insights into novel treatment strategies and diagnostic approaches for this neurodegenerative condition.

In recent years, growing evidence highlighted the role of microglia and its related neuroinflammation in the pathogenesis and progression of AD (S. Hong et al., 2016; Hansen et al., 2018). As the innate immune cells of the central nervous system (CNS), microglia could play a crucial role in nervous system injury response and pathogen defense, as well as the developmental sculpting of neural circuits by engulfment and removal of unwanted neurons and synapses (Ransohoff and Cardona, 2010; Tay et al., 2017; Li and Barres, 2018). It has been reported that activated microglia (De Strooper and Karran, 2016) and aberrant proinflammatory phenotype contribute to neuronal damage and synaptic dysfunction by releasing cytokines, reactive oxygen species, and other inflammatory mediators, which could perpetuate neurodegeneration and exacerbate the progression of AD (Tang and Le, 2016). Moreover, microglial responses to plaque formation have also been considered an important factor in AD pathogenesis (Condello et al., 2015). It has been reported that the impairment of microglial function to reduce Aβ burden is a causal factor in AD. Specifically, microglial activation attempts to eliminate Aβ accumulation by phagocytosis and clearance (Yin et al., 2023); however, the generation and accumulation of Aβ could cause microglial dysfunction by releasing the inflammatory mediators (Sosna et al., 2018).

Triggering receptor expressed in myeloid cells 2 (TREM2) is a cell surface protein selectively and highly expressed by microglia in the brain, which has been proven to maintain the phagocytic ability of microglia toward Aβ deposits (Y. Wang et al., 2015; S. Wang et al., 2022). The results of our previous studies showed that the imbalance of TREM2 expression was not only involved in the process of neuronal injury induced by high cholesterol but also related to LPS-induced hyperactivation of microglia and increased secretion of inflammatory factors (X. R. Gao et al., 2021; Liu et al., 2022; Zheng et al., 2023). Moreover, both T2DM and AD mice models showed a neuroinflammation-related imbalance of TREM1/2 expression in the hippocampus, with a close relation with the impaired performance in behavioral tasks (Fan et al., 2022). Hence, it should be rational to hypothesize the potential role of TREM2 in linking AD and TREM2.

Focusing on the intersection of T2DM and AD, we aim to elucidate the mechanisms by which metabolic dysfunction may contribute to neurodegenerative changes. The T2DM mice model was established by the combination of high-fat diet (HFD) and STZ/I.P. Apart from the metabolic parameters, the behavioral performance was observed by open-field test (OFT), novel object recognition test (NOR), Y-maze test (Y-maze), and Morris water maze test (MWM). The neuron damage (mark proteins with Map-2 and NeuN), the pathological index of AD (mark proteins with APP and p-Tau), and the activation of astrocytes and microglia (mark proteins with GFAP and IBA-1) in the hippocampus were observed by immunofluorescence. The protein expression levels of the insulin signaling pathway including IRS1, Akt, and ERK and AD-related biomarkers including APP, p-Tau, and TREM1/2 in the hippocampus and PFC were detected via Western blot, and the mRNA expression levels of IL-1β, IL-6, TNF-α, ocln, zo-1, and clnds were measured by qPCR technique.

## Materials and Methods

### Animal experimental design and drug treatment

**Figure 1.**
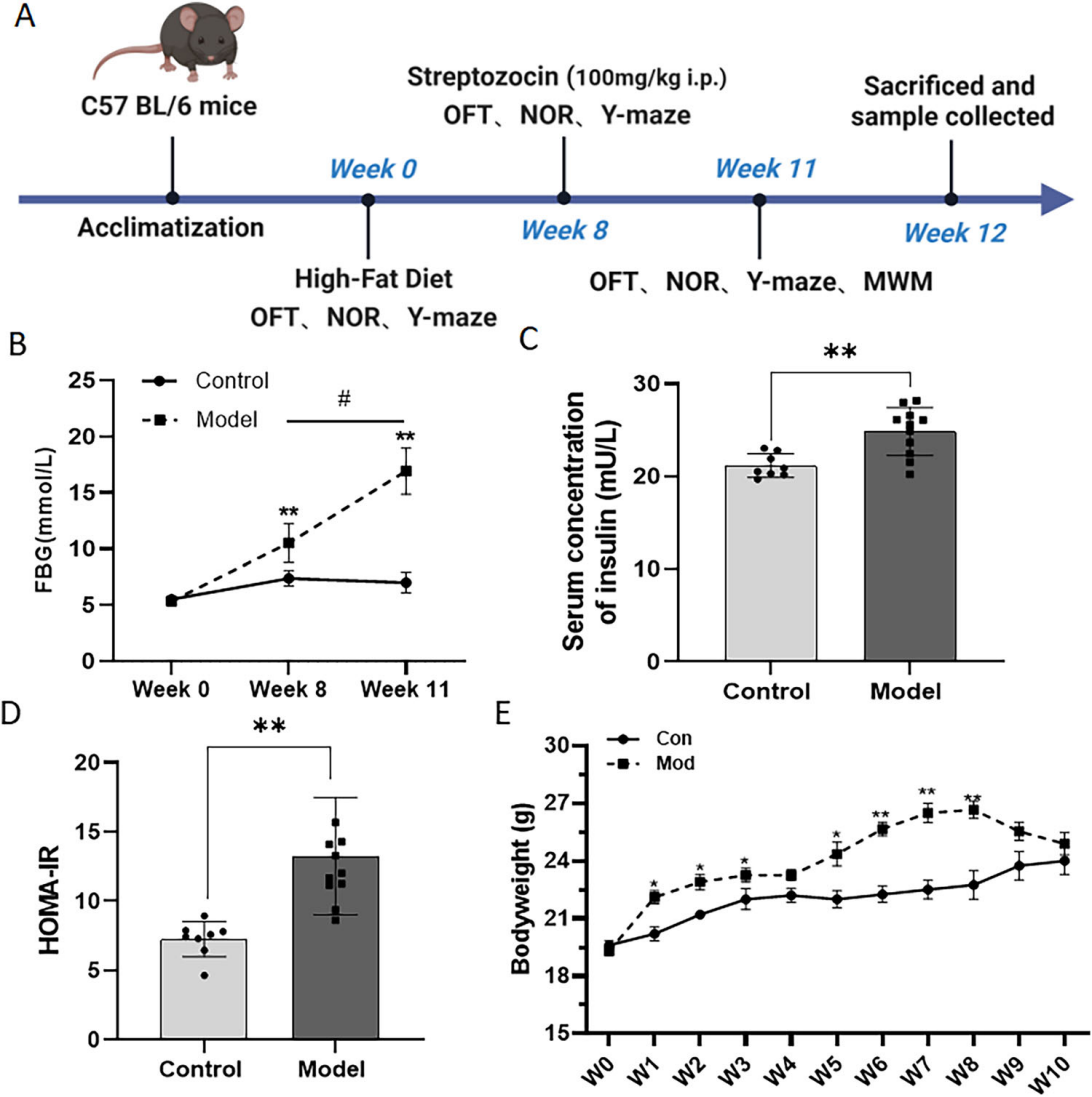
Combination of HFD and STZ/I.P. induced hyperglycemia, hyperinsulinemia, and insulin resistance in mice. ***A***, Schedule of the experimental design. ***B***, The fasting blood glucose (FBG) levels of mice after 12  h of food deprivation. ***C***, The serum concentrations of insulin in mice. ***D***, The HOMA-IR index of mice. ***E***, The body weight of the mice during the experiment. The data are presented as the mean ± SEM, with *n* = 8 mice in each group. *p < 0.05 and **p < 0.01 compared with the Con group.

Sixteen male C57BL/6 mice, aged 6 weeks, were obtained from the Experimental Animal Center of Anhui Medical University. The mice were housed in cages with four mice per cage and subjected to alternating light and dark conditions for 12 h. They had access to adequate food and water, and the ambient temperature was maintained at 20 ± 2°C with a humidity of 50 ± 5%. The study protocol was approved by the Ethics Committee of Experimental Animals of Anhui Medical University and followed the National Institutes of Health Guide for the Care and Use of Laboratory Animals. Measures were implemented to minimize pain and discomfort for the mice throughout the entire experimental process. After 1 week of acclimatization, the mice were randomly divided into two groups, including the control group (Con) fed a standard chow diet (3.766 kcal/g) and the model group (Mod) fed a high-fat diet (60% of calories from fat, 5 kcal/g). After 8 weeks of feeding, the model mice were injected intraperitoneally with STZ (100 mg/kg), while the control mice were injected with a buffer solution. The experimental procedure is depicted in Figure 1*A*.

### Behavioral tests

All behavioral tests were conducted in a quiet laboratory, with testing times scheduled between 08:00 and 12:30 to ensure consistent conditions across all groups. Prior to each experiment, the mice were acclimated to the behavioral laboratory environment to minimize anxiety and hyperactivity. After each experiment, all equipment and utensils were thoroughly cleaned and wiped with 75% alcohol to prevent any residual odors or excrement that could potentially impact the behavior of subsequent mice. The ANY-maze video imaging software (Stoelting) was utilized to record and analyze the performance of the mice in the behavioral tests.

### Open-field test (OFT)

The OFT was conducted to assess the spontaneous exploratory activity of mice, whose apparatus consisted of a white rectangular box measuring 50 × 50× 45 cm. Each mouse was placed in one corner of the box and allowed to freely explore the field for 5 min. The total distance, average speed, and line crossing of mice traveled in the OFT were recorded and analyzed.

### Novel object recognition test (NOR)

The NOR was used to assess the cognitive function of mice, whose apparatus is similar to the OFT with some modifications. The experiment consists of two stages: the adaptation period and the experimental period. During the adaptation period, the mouse was placed into the apparatus for 10 min to familiarize with the environment. The experimental period is divided into two stages: during the first stage, two identical objects were placed on the floor, and the mouse was allowed to freely explore the objects for 10 min; after 1 h, during the second stage, one of the familiar objects was replaced with a novel object, which had a different color and shape but was placed in the same position as the familiar object, and the mouse was then reintroduced into the apparatus and allowed to explore the novel and familiar objects for 5 min. The novel object recognition index was calculated as the ratio of the exploration time the mouse spent on the novel object and the old object.

### Y-maze test (Y-maze)

The Y-maze was conducted to evaluate the spatial memory of mice, whose apparatus consisted of three interconnected arms (40 × 10 × 20 cm) arranged radially at 120° angles from one another. The arms were randomly designated as the start, familiar, or novel arm. During the adaptation period, the novel arm was blocked off, and the mouse was placed from the start arm and allowed to explore the apparatus for 10 min. One hour later, with the novel arm open, the mouse was placed back and allowed to explore the three arms for 5 min freely. The novel arm preference index was calculated as the ratio of duration the mouse spent in the novel arm and the familiar arm.

### Morris water maze test (MWM)

The MWM was utilized to assess the spatial memory of mice, whose apparatus consisted of a black circular pool with a diameter of 120 cm and a height of 80 cm. The water temperature was maintained at 21–23°C and colored white using food coloring. The maze was divided into four equal quadrants: the first, second, third, and fourth quadrants. A circular platform, positioned 1 cm below the water surface, was placed in one of the quadrants and designated as the target quadrant. The test comprised two stages: the acquisition phase and the probe test. During the acquisition phase, the mouse was initially allowed to adapt to the platform’s location by spending 30 s on it. Subsequently, the mouse was placed in the water, facing the wall, in each quadrant and trained to locate the hidden platform within 60 s. This training was repeated for 3 consecutive days, and the average time the mouse spent in four trials per day was recorded as the escape latency. During the probe test, the submerged escape platform was removed, and the mouse was placed into the pool opposite to the platform and allowed to swim freely for 60 s. The time mice spent in the target quadrant and the latency to the target quadrant were recorded and analyzed.

### Measurement of serum samples

The blood glucose levels were measured using a Roche glycemic meter. Twenty-four hours after the last behavioral test, the mice were deeply anesthetized after an overnight fast. Blood was collected and placed at room temperature for 2 h. The serum was isolated by centrifugation at 3,000 rpm for 15 min, and the upper layer was collected. The serum levels of insulin were measured using commercially available enzyme-linked immunosorbent assay (ELISA) kits (Wuhan ColorfulGene Biological Technology). The HOMA-IR index was calculated using the following formula: HOMA-IR = (fasting glucose (mmol/L) × fasting insulin (mU/L)) / 22.5.

### Immunofluorescence staining (IF)

Three mice were randomly selected and treated with a perfusion of PBS and 4% paraformaldehyde until systemic spasm occurred. Then the whole brains were fixed overnight in a 4% paraformaldehyde solution and dehydrated in a 30% sucrose solution for 48 h. Subsequently, the brains were embedded in an OCT embedding agent (4583, Solarbio) and frozen at −80°C. Brain slices were cut for 30 µm using a cryostat. After antigen retrieval with EDTA in a 95°C water bath, the sections were blocked with 5% BSA and 0.3% Triton X-100 at room temperature for 1 h. Following the blocking step, the sections were incubated overnight at 4°C with primary antibodies: anti-APP (1:100; 25524-1-AP, Proteintech), anti-p-Tau (Ser396; 1:100; sc-32275, Santa Cruz Biotechnology), anti-GFAP (1:100; 16825-1-AP, Proteintech), anti-IBA1 (1:100; DF6442, Affinity Biosciences), anti-CD68 (1:100, 28058-1-AP, Proteintech), anti-NeuN (1:100; ET1602-12, Huabio), and anti-Map-2 (1:100; EM1709-48, Huabio). Then the sections were incubated with either goat anti-rabbit Alexa Fluor 488–conjugated IgG (1:100; ZF-0511, ZSGB-BIO) or goat anti-mouse Alexa Fluor 647–conjugated IgG (1:100; A0473, Time) at room temperature for 2 h. Finally, nuclei were counterstained with DAPI. The stained slices were observed under a fluorescence microscope (Olympus Life Science VS120), and fluorescence intensity was quantified using Image-Pro software (Media Cybernetics).

### Western blotting

Three mice were randomly selected from each group, and the hippocampus and the PFC were carefully separated, rapidly frozen in liquid nitrogen, and stored at −80°C. Then, the hippocampus and PFC tissues were cleaved using RIPA buffer (P0013B, Beyotime) containing protease inhibitors (ST507-10, Beyotime) and phosphatase inhibitors (ST019-10, Beyotime). Equal amounts of protein were separated on 10% SDS–PAGE gels, transferred onto polyvinylidene difluoride (PVDF) membranes, and incubated overnight at 4°C with the relevant primary antibodies: anti-phosphorylated AKT (p-AKT) and total AKT (1:1,000; Zen-Bio), anti-phosphorylated ERK (p-ERK) and total ERK (1:1,000; Santa Cruz Biotechnology), anti-phosphorylated IRS1 (p-IRS1) and total IRS1 (1:1,000; Abcam), anti-APP(1:1,000; Proteintech), anti-phosphorylated Tau (p-Tau) and total tau (1:1,000; Santa Cruz Biotechnology), anti-TREM1 and TREM2 (1:1,000; Abcam), and anti-β-actin (1:1,000; Zhongshan Biotechnology). After washing with TBST for 30 min, the membranes were incubated with corresponding secondary antibodies (HRP-conjugated anti-rabbit or anti-mouse antibodies) at room temperature for 1 h. In addition, in order to detect multiple targets, the Western blot fast stripping buffer (PS107, Epizyme) was used due to the similar molecular weight of the target bands. Protein images were captured using a gel imaging system, and the images were processed and analyzed using ImageJ software (National Institutes of Health) and normalized relative to that of the internal control β-actin.

### Quantitative real-time PCR

Equal quantities of total RNA from hippocampal and PFC tissues were used for qPCR analysis. Briefly, the total RNA was extracted using the TRIzol reagents and transcribed into cDNA using commercial kits according to the instructions. The reverse transcription reaction was performed at 37°C for 15 min and 85°C for 5 s. The mRNA expression levels of ocln, zo1, clnds, IL-1β, LI-6, and TNF-α in the hippocampus and PFC of mice were detected using the SYBR Green PCR kit on an ABI Prism Sequence Detector System in a 20 µl volume for 40 cycles (10 s at 95°C and 30 s at 55°C). Three replicates were conducted for each qPCR analysis, and the data were analyzed using the 2^−ΔΔCt^ method. The primer sequences used in the PCR are provided in Table 1.

**Table 1.** Primer sequences

Gene	Base sequence (5′−3′)
β-Actin	F: 5′-AGTGTGACGTTGACATCCGT-3′
	R: 5′-TGCTAGGAGCCAGAGCAGTA-3′
TNF-α	F: 5′-CACCACCATCAAGGACTCAA-3′
	R: 5′-AGGCAACCTGACCACTCTCC-3′
IL-1β	F: 5′-CTTTGAAGTTGACGGACCC-3′
	R: 5′-TGAGTGATACTGCCTGCCTG-3′
IL-6	F: 5′-GAGGATACCACTCCCAACAGACC-3′
	R: 5′-AAGTGCATCATCGTTGTTCATACA-3′
Ocln	F: CTGGATCTATGTACGGCTCACA
	R: TCCACGTAGAGACCAGTACCT
ZO-1	F: ACCACCAACCCGAGAAGAC
	R: CAGGAGTCATGGACGCACA
Clnd5	F: ACTCTTTGTTACCTTGACCGG
	R: CAGCTCGTACTTCTGTGACAC

### Statistical analysis

All experimental data were analyzed and visualized using SPSS Statistics 17.0 software, and the charts were drawn using GraphPad Prism 8.0 software. The differences between the two groups were assessed using the Student’s *t* test. Although the differences between the two groups were initially assessed using the Student's *t* test, we also conducted the Wilcoxon rank-sum test to account for potential deviations from normality and small sample size. For comparisons of more than two groups, two-way (repeated measures) ANOVA was used to assess statistical significance, followed by multiple comparisons between groups using Bonferroni’s post hoc test. Data are presented as mean ± standard error of the mean (SEM), and *p* < 0.05 was considered statistically significant. All data points were included in the plots to maintain transparency and facilitate a comprehensive analysis of the results.

## Results

### Combination of HFD and STZ/I.P. induced hyperglycemia, hyperinsulinemia, and insulin resistance in mice

According to the longitudinal epidemiological studies, there have been elevated plasma insulin levels (Craft et al., 1998) in AD patients, with a close association between cognitive disorder and insulin resistance in T2DM patients (Arnold et al., 2018; Kshirsagar et al., 2021). Similarly, in the present study, the mice in the Mod group exhibited a significant increased blood glucose levels and serum levels of insulin when compared with the Con group, as shown in Figure 1*B,C*. Furthermore, the results showed that the HOMA-IR index was significantly increased in the Mod mice as compared with the Con mice, as shown in Figure 1*D*. The tendency of the bodyweight in the Mod group was increased after HFD induction and began to decline upon STZ treatment after 8 weeks when compared with the Con group in Figure 1*E*.

### Combination of HFD and STZ/I.P. induced an AD-like behavioral dysfunction in mice

**Figure 2.**
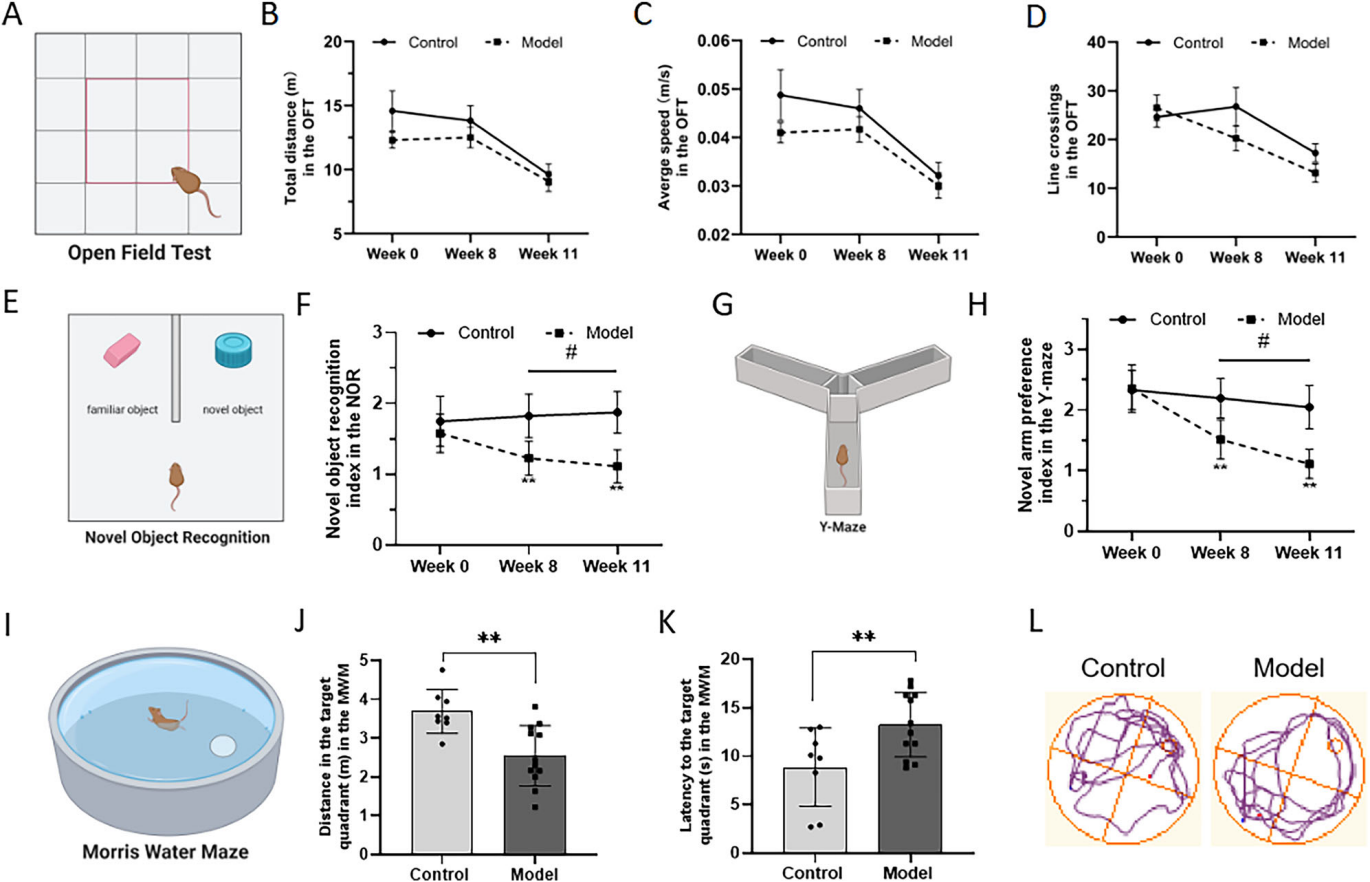
Combination of HFD and STZ/I.P. induced AD-like neuropsychiatric dysfunction in mice. ***A***, Outline of the OFT procedure. ***B***, Total distance in the OFT. ***C***, Average speed in the OFT. ***D***, Line crossing in the OFT. ***E***, Outline of the NOR procedure. ***F***, The novel object recognition index in the NOR. ***G***, Outline of the Y-maze procedure. ***H***, The novel arm preference index in the Y-maze. ***I***, Outline of the MWM procedure. ***J***, The distance in the target quadrant and (***K***) the latency to the target quadrant in the MWM. ***L***, The typical moving orbits in the MWM. The data are presented as the mean ± SEM, with *n* = 8 mice in each group. *p(^#^*p) *< 0.05 and **p(^##^*p)* < 0.01 compared with the Con group.

The general behavior of mice is shown in Figure 2. As shown in Figure 2*A,D*, there was no significant difference between the two groups in the total moving distance, average speed, and line crossing in the OFT. The behavioral performance of mice in the NOR and Y-maze test are shown in Figure 2*E–H*. Compared to the Con mice, the Mod mice exhibited a significant decrease in the novel object recognition index (Fig. 2*E,F*), together with a decreased novel arm preference index (Fig. 2*G,H*). Moreover, in the MWM test, the Mod mice showed a decreased distance traveled in the target quadrant and an increased latency found in the target quadrant in the probe trial (Fig. 2*I–K*). The typical swimming orbits of the two groups in the probe trial are shown in Figure 2*L*.

### Combination of HFD and STZ/I.P. induced neuron injuries and blood–brain barrier damage in the hippocampus in mice

**Figure 3.**
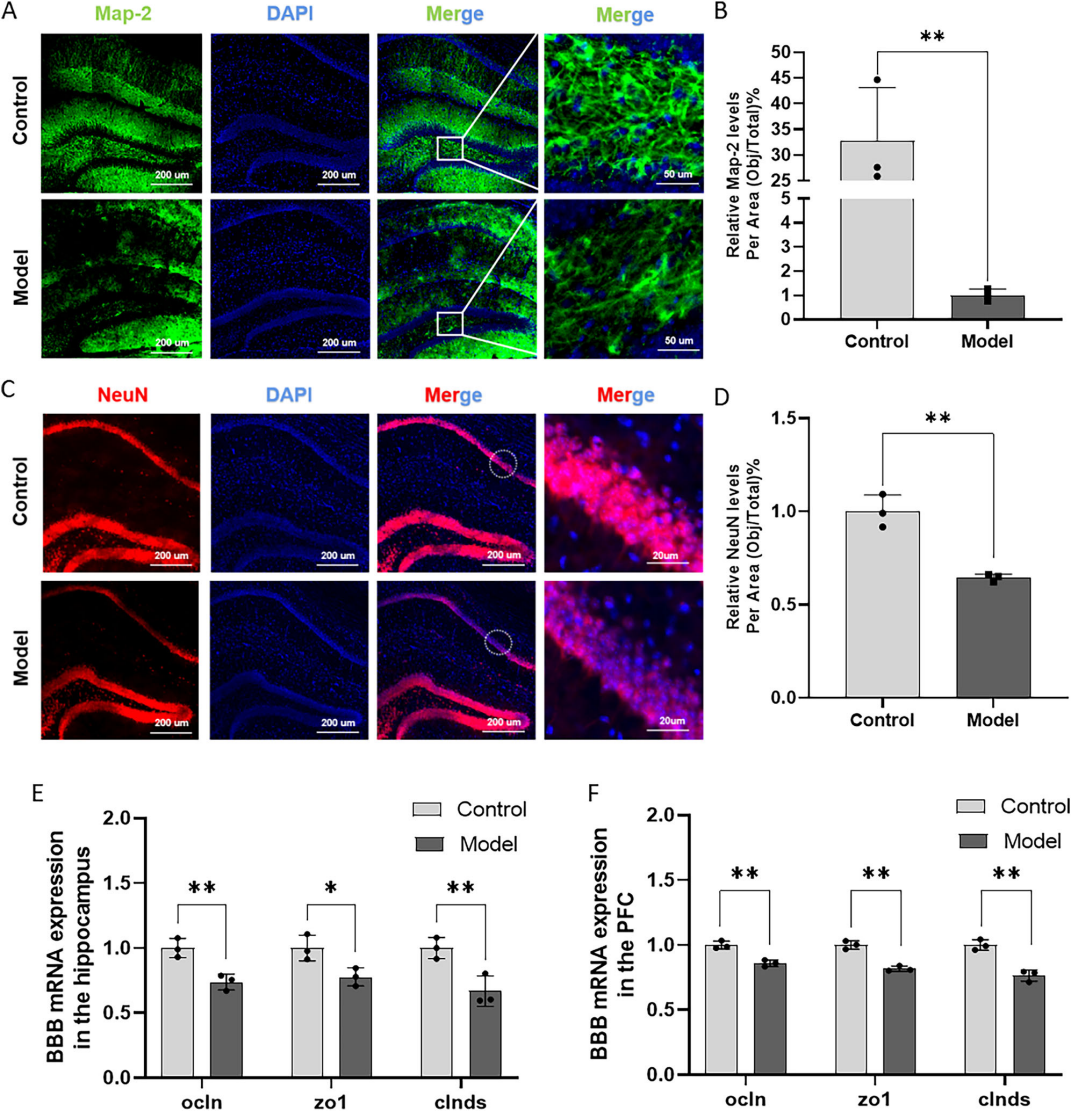
Combination of HFD and STZ/I.P. induced neuron injuries and blood–brain barrier damage in the hippocampus in mice. ***A***, The typical immunofluorescent images of Map-2 (green) in the hippocampus of mice. Scale bar, 200 or 50 μm. ***B***, Quantifcation of the pixels of Map-2–positive area. ***C***, The typical immunofluorescent images of NeuN (red) in the hippocampus of mice. Scale bar, 200 μm. ***D***, Quantifcation of the pixels of NeuN-positive area. ***E***, ***F***, The mRNA expression levels of ocln, zo-1, and clnds in the hippocampus and PFC of mice. The data are presented as the mean ± SEM, with *n* = 3 in each group. *p < 0.05 and **p < 0.01 compared with the Con group.

Map-2 and NeuN are the neuronal marker proteins used to assess the morphology and function of neurons, and detecting the expression of Map-2 and NeuN could help know the extent of neuronal damage and degeneration in the present study (Götz and Ittner, 2008; Ghatak et al., 2019; Sattarov et al., 2023). As shown in Figure 3*A,B*, the Mod group exhibited significantly lower fluorescence intensity for Map-2 in the hippocampal region, suggesting a reduction in the expression of this neuronal marker compared with the control group. Similarly, the immunofluorescent staining of NeuN was also reduced in the hippocampus of Mod mice as compared with the Con mice (Fig. 3*C,D*), indicating that combination of HFD and STZ/I.P. could induce neuron damage in the hippocampus of mice. Besides, the mRNA expression levels of ocln, zo-1, and clnds in the hippocampus and PFC of Mod mice were all remarkably reduced compared with the Con ones, suggesting potential BBB damage induced by the combination of HFD and STZ/I.P. in mice (Fig. 3*E,F*).

### Combination of HFD and STZ/I.P. induced Aβ accumulation and tau hyperphosphorylation in mice

**Figure 4.**
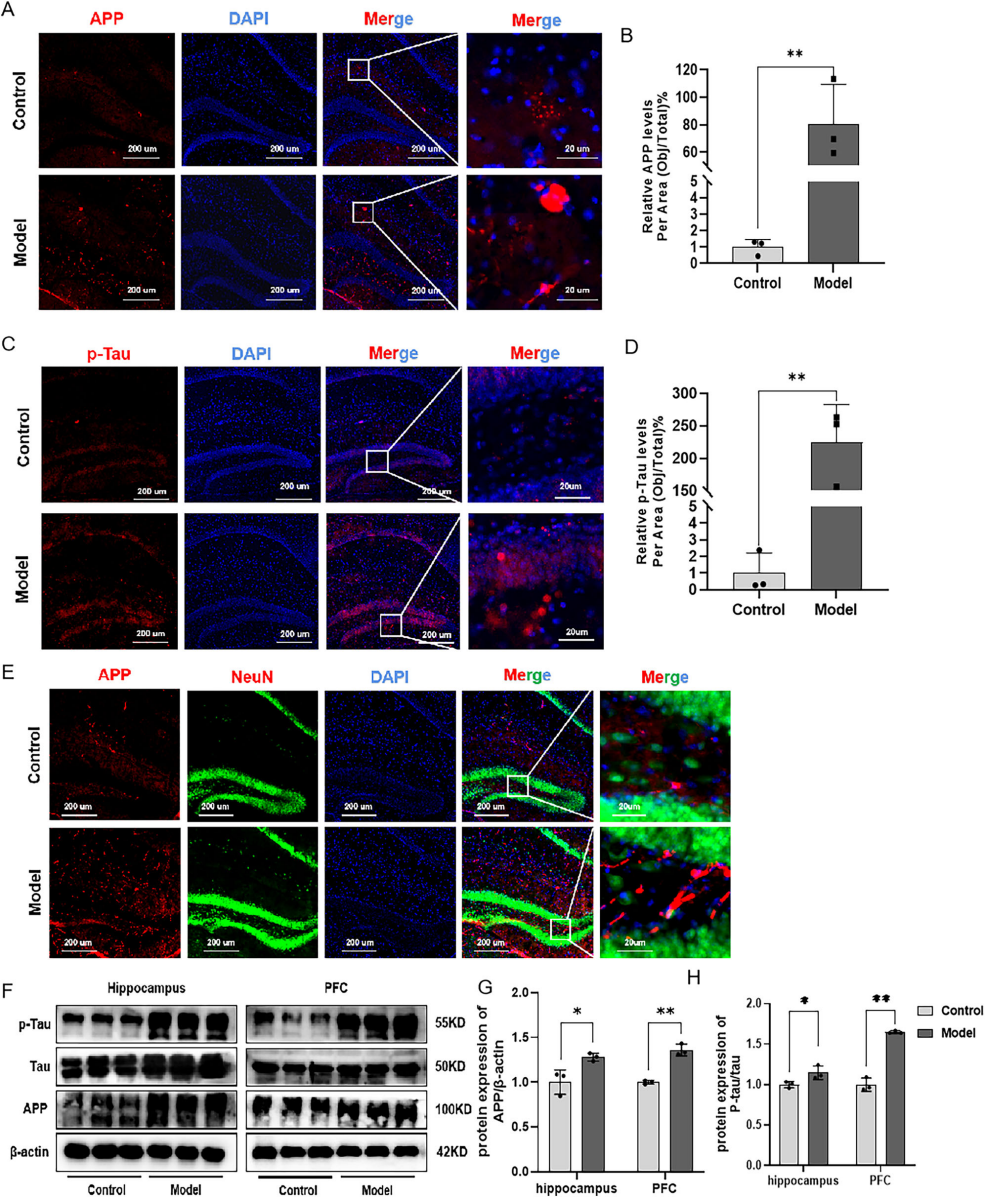
Combination of HFD and STZ/I.P. induced APP accumulation and tau hyperphosphorylation in mice. ***A***, ***C***, The typical immunofluorescent images of APP (red) and p-Tau (red) in the hippocampus of mice. Scale bar, 200 or 50 μm. ***B***, ***D***, Quantification of the pixels of APP and p-Tau–positive area. ***E***, The colocalization of Aβ with neurons. ***F***, The typical graph of p-Tau and APP proteins in the hippocampus and PFC of mice. ***G***, ***H***, The statistical analysis of the Western blotting results in the hippocampus and PFC. The data are presented as the mean ± SEM, with *n* = 3 in each group. *p < 0.05 and **p < 0.01 compared with the Con group.

As shown in Figure 4*A,B*, compared with the Con mice, there has been a significant increase in APP immunofluorescence intensity in the hippocampus of Mod mice. Furthermore, the immunofluorescent staining of p-Tau was also increased in the hippocampus of Mod mice as compared with the Con mice (Fig. 4*C,D*). We also conducted a colocalization of APP and neuronal marker (Fig. 4*E*). Similarly, as shown in Figure 4*F,H*, the protein expression levels of APP, p-Tau, and Tau in the hippocampal and PFC tissues of mice in the Mod group were significantly increased compared with the Con ones, suggesting that combination of HFD and STZ/I.P. could induce Aβ accumulation and tau hyperphosphorylation in the hippocampus and PFC of mice.

### Combination of HFD and STZ/I.P. promoted the activation of microglia and astrocytes in mice

**Figure 5.**
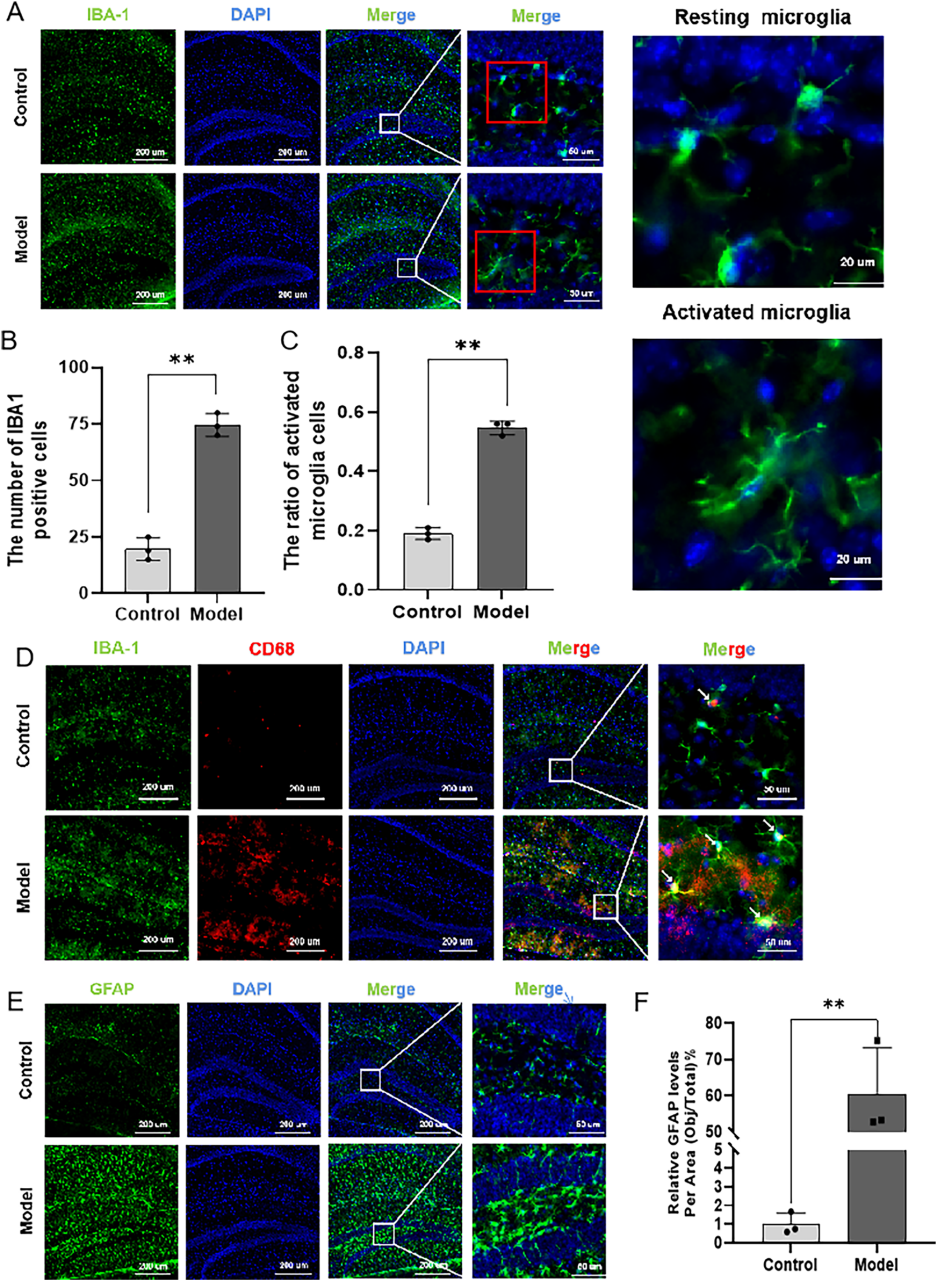
Combination of HFD and STZ/I.P. promoted the activation of microglia and astrocytes in mice. ***A***, The typical immunofluorescent images of IBA-1 (green) in the hippocampus of mice. Scale bar, 200, 50, or 20 μm. ***B***, Quantification of the number of IBA1 positive cells. ***C***, The ratio of activated microglia to the total number of microglia. ***D***, The colocalization of CD68 with IBA-1. ***E***, The typical immunofluorescent images of GFAP (green) in the hippocampus of mice. Scale bar, 200 or 50 μm. ***F***, Quantification of the pixels of GFAP-positive area. The data are presented as the mean ± SEM, with *n* = 3 in each group. *p < 0.05 and **p < 0.01 compared with the Con group.

As shown in Figure 5*A–D*, compared with the Con mice, the number of IBA-1–positive cells (Fig. 5*B*) and the ratio of activated microglia cells to the total number of microglia cells (Fig. 5*C*) were both significantly increased in the hippocampus of Mod mice. Additionally, the Mod group demonstrated a higher prevalence of CD68+ microglia than the Con group (Fig. 5*D*). Representative images of different states of microglial cells are shown in Figure 5*A*. Furthermore, the immunofluorescent staining of GFAP-positive cells was also increased in the hippocampus of Mod mice as compared with the Con mice (Fig. 5*E,F*), suggesting that combination of HFD and STZ/I.P. could promote the activation of microglia and astrocytes in the hippocampus of mice.

### Combination of HFD and STZ/I.P. induced the dysregulation of the IRS1/AKT/ERK signaling pathway in the hippocampus and the PFC in mice

**Figure 6.**
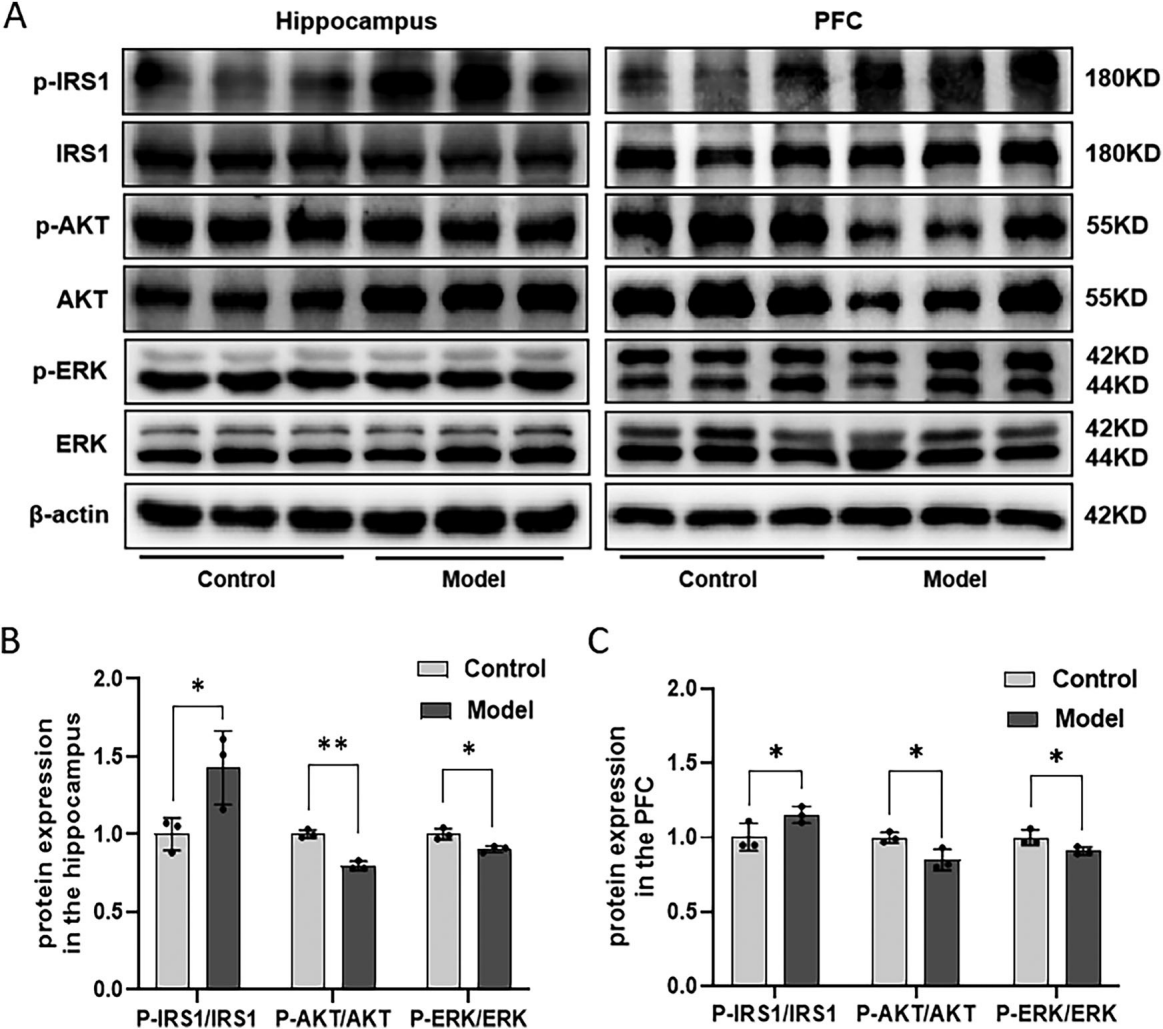
Combination of HFD and STZ/I.P. induced the dysregulation of the IRS1/AKT/ERK signaling pathway in mice. ***A***, The typical graph of IRS1/AKT/ERK signaling pathway proteins in the hippocampus and PFC of mice. ***B***, The statistical analysis of the Western blotting results in the hippocampus. ***C***, The statistical analysis of the Western blotting results in the PFC. The data are presented as the mean ± SEM, with *n* = 3 in each group. *p < 0.05 and **p < 0.01 compared with the Con group.

Figure 6 shows the expression of proteins in the IRS1/AKT/ERK signaling pathway in the hippocampus and PFC of the mice. Compared with that in the Con group, the relative protein expression of p-IRS1/IRS1 was increased in the hippocampus and PFC of Mod mice, while the relative protein expression of p-AKT/AKT and p-ERK/ERK were both decreased in the hippocampus and PFC of Mod mice when compared with the Con mice (Fig. 6*A–C*), suggesting that combination of HFD and STZ/I.P. could induce the dysregulation of the IRS1/AKT/ERK signaling pathway in the hippocampus and the PFC of mice.

### Combination of HFD and STZ/I.P. induced the imbalance of TREM1/2 and increased inflammatory factors in the hippocampus and the PFC in mice

**Figure 7.**
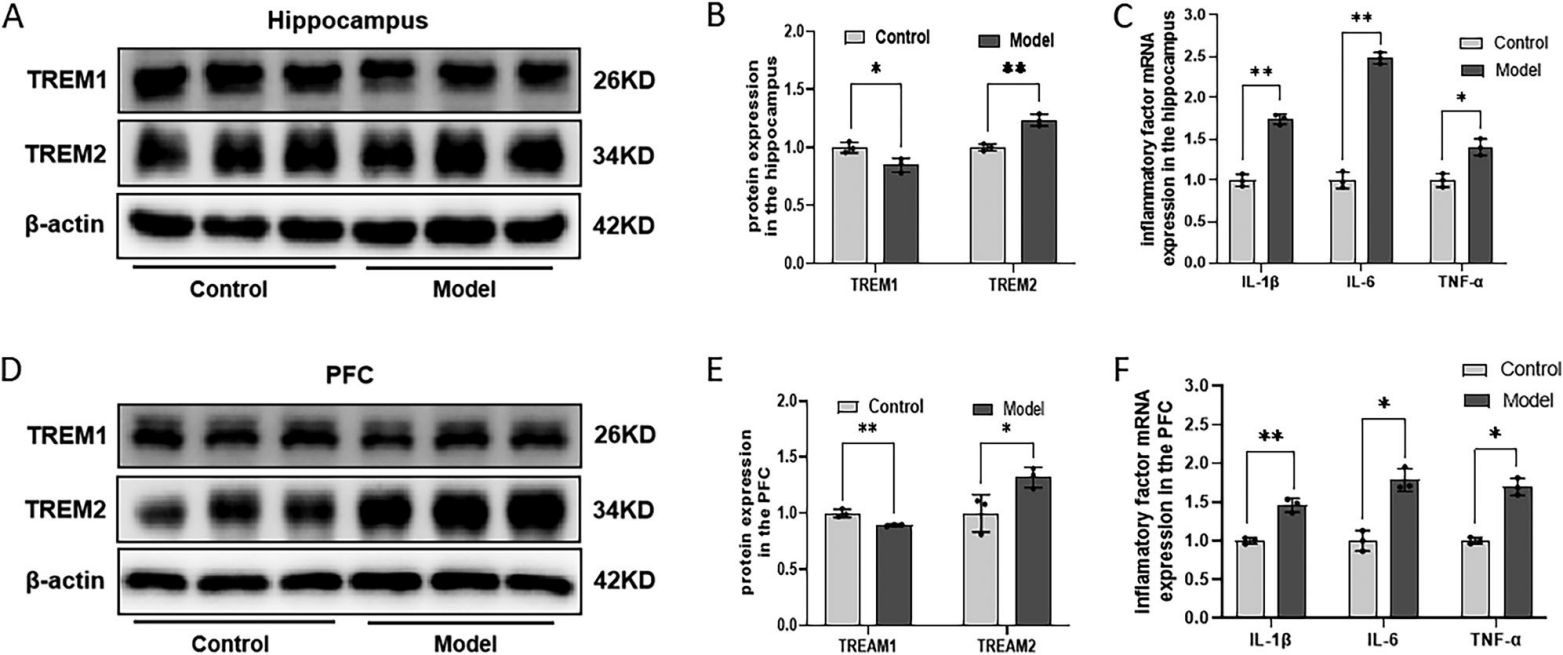
Combination of HFD and STZ/I.P. induced the imbalance of TREM1/2 and increase of inflammatory factors in the hippocampus and the PFC of mice. ***A***, ***D***, The typical graph of TREM1 and TREM2 in the hippocampus and PFC of mice. ***B***, ***E***, The statistical analysis of the Western blotting results in the hippocampus and PFC. ***C***, ***F***, The mRNA expression levels of IL-1β, IL-6, and TNF-α in the hippocampus and PFC of mice. The data are presented as the mean ± SEM, with *n* = 3 in each group. *p < 0.05 and **p < 0.01 compared with the Con group.

Figures 7, *A, D*, and *E*, and 8*B* show the expression of TREM1 and TREM2 in the hippocampus and PFC of the mice. Compared to the Con mice, the Mod mice exhibited a significant decrease in the protein expression of TREM1 and an obvious increase in the protein expression of TREM2 in both the hippocampus (Fig. 7*A,B*) and PFC (Fig. 7*D,E*), indicating an imbalanced expression of TREM1/2 induced by the combination of HFD and STZ/I.P. in mice. Additionally, as depicted in Figure 7*C,F*, the mRNA expression levels of the inflammatory factors IL-1β, IL-6, and TNF-α in the hippocampus and PFC of Mod mice were all remarkably increased as compared with the Con ones, suggesting an increased inflammatory response induced by the combination of HFD and STZ/I.P. in mice.

**Figure 8.**
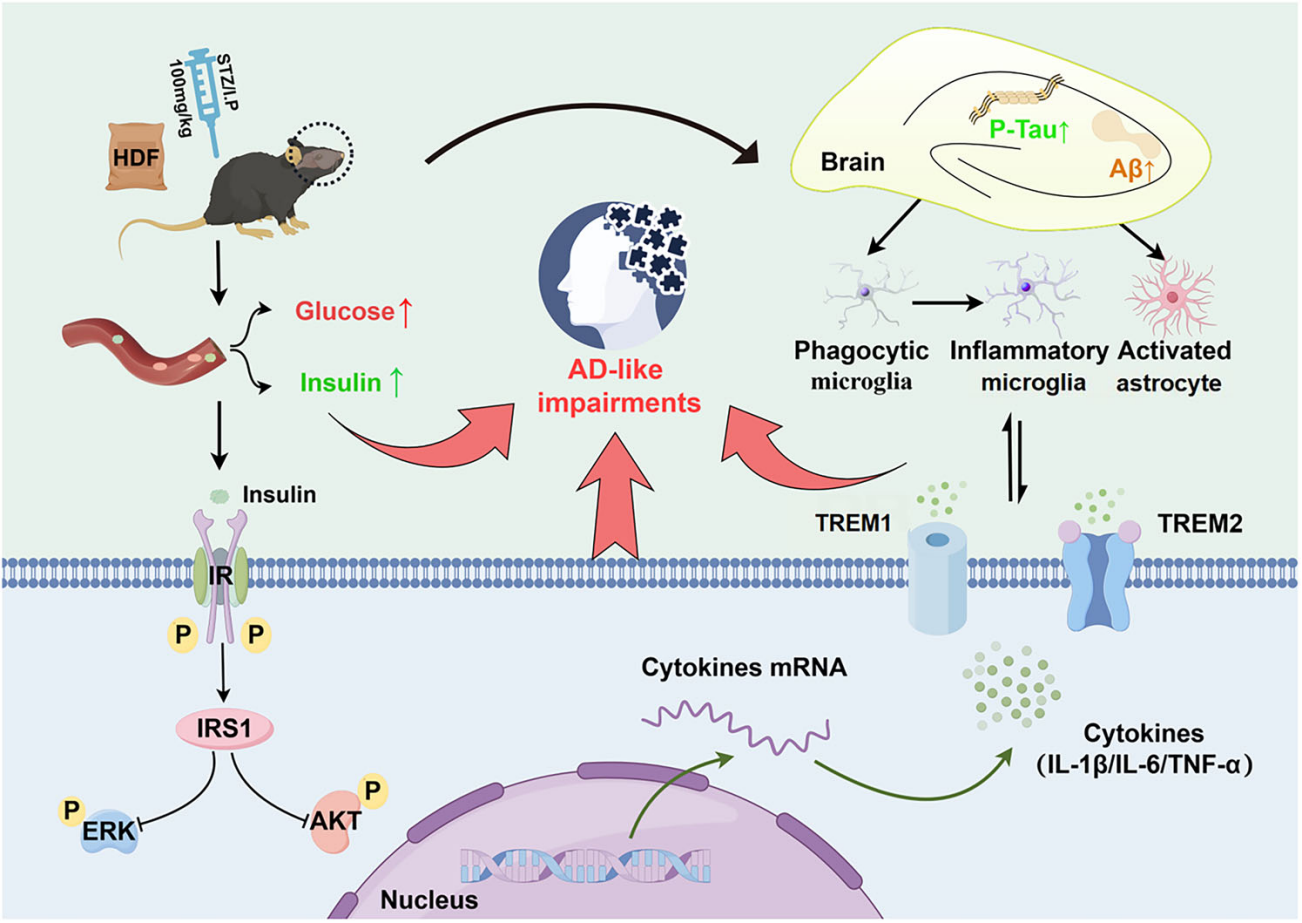
AD-like neuropsychiatric dysfunction in a mice model induced by the combination of high-fat diet and intraperitoneal injection of streptozotocin. Apart from the Aβ accumulation and tau hyperphosphorylation, the neuron damage and activation of microglia and astrocytes in the hippocampus, as well as the imbalanced protein expression of the IRS1/AKT/ERK signaling pathway and TREM1/2, may also be involved in AD-like pathological process.

## Discussion

In the present study, the AD-like neuropsychiatric dysfunction, both the behavioral performance and the neuropathological changes, was investigated in a mice model induced by the combination of HFD and STZ/I.P. The results showed that combination of HFD and STZ/I.P. could induce AD-like metabolic disorders and behavioral impairments in mice, including increased blood glucose levels and serum insulin levels and and impaired cognitive ability in NOR, Y-maze, and MWM tests. Moreover, compared with the Con group, combination of HFD and STZ/I.P. could also induce neuron damage and activation of microglia and astrocytes in the hippocampus of mice, as well as the APP accumulation, tau hyperphosphorylation, imbalanced protein expression of the IRS1/AKT/ERK signaling pathway and TREM1/2, and increased inflammatory factors in the hippocampus and PFC of mice.

Hyperglycemia and insulin resistance, which are typical clinical features of T2DM, have also been increasingly recognized as an important metabolic index in the pathogenesis of AD (Grodstein et al., 2001; Luchsinger et al., 2004). It has been reported that chronic hyperglycemia could promote the formation of advanced glycation end products and oxidative stress (Goh and Cooper, 2008; Joubert et al., 2019), and insulin resistance could affect neuronal function and Aβ accumulation in the brain, which contribute to neuroinflammation and synaptic dysfunction and finally lead to cognitive decline (Arnold et al., 2018). Given the killing effect of STZ on the pancreatic β-cell, it has been widely used in establishing T2DM animal models (Ganda et al., 1976; Grieb, 2016). Moreover, besides its efficiency in inducing T2DM-like metabolic disorders including hyperglycemia and hyperlipidemia, the results of our previous studies suggested that STZ could induce behavioral impairments in rodents (Qi et al., 2021; Fan et al., 2022; X. Gao et al., 2023b). Consistently, in the present study, our results showed that combination of HFD and STZ/I.P. could induce a significant increase in blood glucose level, serum insulin, and the HOMA-IR index in mice. Moreover, the model mice showed decreased learning and memory ability in the NOR, Y-maze, and MWM tasks, although there was no significant difference between groups in the behavioral performance in the OFT. These results suggested that the combined use of HFD and STZ/I.P. could successfully induce AD-like metabolic and behavioral injuries in mice.

The hippocampus is a vital brain structure that serves as a critical hub for cognitive processes, playing a central role in learning function, memory consolidation, and spatial navigation (Preston and Eichenbaum, 2013). Research studies have shown that hippocampal structural abnormalities and neuronal loss are the key neuropathological hallmarks of AD, contributing to the cognitive decline observed in the disease (Venkateshappa et al., 2012; Scheltens et al., 2021). The microtubule-associated protein 2 (Map-2) and neuronal nuclei (NeuN) are widely used in marking the mature neurons and neuronal nuclei in the brain (Götz and Ittner, 2008). In this study, compared with the control group, immunofluorescence staining of Map-2 and NeuN in the hippocampal tissue of Mod mice exhibited a significant reduction, indicating that the combined effect of HFD and STZ/I.P. can induce hippocampal neuronal damage in mice, which is consistent with our previous findings (X. Gao et al., 2023b).

The most significant pathological changes in Alzheimer's disease are the accumulation of abnormal protein aggregates in the brain, primarily including Aβ plaques and tau neurofibrillary tangles. These abnormal aggregates lead to neuronal dysfunction, synaptic damage, and neurodegeneration in Alzheimer's disease patients (Scheltens et al., 2016, 2021). Studies have shown that Aβ aggregation and excessive phosphorylation of tau can impair microtubule stability, thereby affecting axonal transport, leading to neuronal degeneration and synaptic loss, and exacerbating the progression of cognitive impairment in AD (Selkoe and Hardy, 2016; Jack et al., 2018). In the present study, the results of Western blot and immunofluorescence staining both indicated that the expression levels of Aβ and p-Tau were significantly increased in the hippocampus and PFC of Mod mice as compared with the Con mice. These results indicated that combination of HFD and STZ/I.P. could induce an AD-related pathology in mice, which was in line with the findings in our previous study.

In addition to Aβ and NFTs, growing evidence has highlighted the role of neuroinflammation in the pathogenesis and progression of AD (Medeiros et al., 2010). Astrocytes and microglia are the two important regulatory factors in neuroinflammatory responses. Microglia, accounting for ∼20% of the brain's glial cells, are the immune cells of the CNS and play a critical role in monitoring the brain and maintaining homeostasis by clearing damaged neurons, plaques, and pathogens (Prinz et al., 2019). However, excessive activation or dysfunction of microglia may lead to neurotoxicity, which also could be found around amyloid plaques in the brains of AD patients (Tang and Le, 2016). In addition, astrocytes also play an important role in maintaining brain structure and function, which could undergo morphological and functional changes and transform into reactive astrocytes under pathological conditions. It has been reported that reactive astrocytes are commonly found in postmortem brain tissues of AD patients in areas with high Aβ or tau pathology (Bussian et al., 2018). Consistently, the results of our present study showed that the number of activated microglia cells and the number of astrocyte cells were both significantly increased in the hippocampus of Mod mice as compared with the Con mice, indicating that combination of HFD and STZ/I.P. could promote the activation of microglia and astrocytes in the hippocampus of mice.

Reactive microglia and astrocytes could promote neuroinflammation by releasing cytokines and inflammatory factors such as IL-1β and IL-6 in AD (H. S. Hong et al., 2003). It has been reported that combined stimulation of IL-1β, IL-6, and IFN-γ in U373 glioblastoma cell lines and primary human astrocytes could induce APP production and lead to increased Aβ levels (Blasko et al., 2000). In addition, IL-1β is the key proinflammatory cytokine associated with age-related cognitive decline, and growing evidence has suggested that synaptic plasticity, learning, and memory are more susceptible to IL-1β–induced impairment, particularly in the aging brain (Prieto et al., 2015). Consistently, the results of our previous study also showed increased mRNA and protein expression levels of IL-1β, IL-6, and TNF-α in AD or T2DM mice and oleic acid or palmitic acid (PA) induced BV2 cells (Fan et al., 2022; X. Gao et al., 2023a). Similarly, in the present study, the expression levels of the inflammatory factors IL-1β, IL-6, and TNF-α in the hippocampus and PFC of Mod mice were all remarkably increased, suggesting an increased inflammatory response induced by the combination of HFD and STZ/I.P. in mice.

Recent studies have suggested a potential link between AD and insulin resistance, which exhibits a decreased level of key insulin signaling proteins and an increased level of insulin resistance markers in the brains of AD mice (Craft et al., 1998; Arnold et al., 2018; Kshirsagar et al., 2021). Insulin receptor substrate 1 (IRS1), protein kinase B (AKT), and ERK are the key components of the insulin signaling pathway; impaired IRS1 signaling leads to decreased activation of AKT and subsequent dysregulation of ERK involved in insulin resistance and metabolic dysfunction (Ding et al., 2019). Of note, activated AKT and ERK were also suggested to be the major kinase for tau phosphorylation and APP deposition in AD, and the brain insulin resistance emerging in AD further induces the decreased phosphorylation levels of AKT and ERK (Oliveira et al., 2021). Consistent with this, the results of our previous study demonstrated a decreased expression level of phosphorylated Akt and ERK in PA-induced HT-22 cells, and an imbalanced expression of PI3K/Akt and ERK pathways was also presented in the hippocampus of T2DM mice through DESeq screening (X. Gao et al., 2023a). In line with these findings, the results of the present study showed that the protein expression levels of p-IRS1/IRS1 were increased, while the levels of p-AKT/AKT and p-ERK/ERK were decreased in the hippocampus and PFC of mice induced by the combination of HFD and STZ/I.P., which provides additional evidence linking insulin resistance and AD.

TREM1/2 are the members of the immunoglobulin superfamily involved in the regulation of inflammation and immune responses. It has been reported that there has a close association between genetic polymorphisms of TREM1/2 and the pathogenesis of AD (Y. Wang et al., 2015). Knocking out TREM1 in the brains of APP/PSEN1 mice has been shown to increase Aβ_1–42_ levels and total amyloid plaque burden, and selective overexpression or activation of TREM1 on microglia cells could improve Aβ neuropathology and rescue AD-related spatial cognitive impairments (Jiang et al., 2016). TREM2-expressing microglia cells have been found in the peripheral regions of amyloid plaques in APP23 transgenic (Tg) mice, and increased TREM2 expression is consistent with the progression of amyloid deposition (Frank et al., 2008). Moreover, the knockdown of TREM2 could alleviate the neuroinflammation and prevent neurodegeneration of tau pathology in TREM2^−/−^PS mice compared with wild-type mice (Leyns et al., 2017). The results of our previous studies showed that the imbalance expression of TREM2 was not only involved in the neuronal injury induced by high cholesterol but also related to LPS-induced homeostatic imbalance of microglia and increased secretion of inflammatory factors (Liu et al., 2022; Zheng et al., 2023). Moreover, the neuroinflammation-related imbalance of TREM1/2 expression was also found in the hippocampus of AD and T2DM mice model, with a closely related to the glyeolipid metabolism disorder and cognitive impairment (Fan et al., 2022; X. Gao et al., 2023b). Similarly, the present study also demonstrates a significantly decreased TREM1 expression and increased TREM2 expression in the hippocampus and PFC of mice induced by the combination of HFD and STZ/I.P. Together with the increased activation of microglia and astrocytes, as well as the elevated expression of IL-1β, IL-6, and TNF-α, these results further support the crucial role of neuroinflammation in the pathogenesis of AD.

There are also some limitations in this study. Firstly, the mouse model used in this study may not fully represent the complexity of human AD. Animal models could only partially replicate the disease processes seen in humans, and the findings may not directly translate to human patients. Secondly, the present study identified several potential mechanisms, and further research is needed to elucidate the underlying pathways and their interactions. Additionally, the role of other factors such as genetic predisposition and environmental influences in the development of AD-like neurophysiological features in T2DM needs to be further explored. Thirdly, the sample size in the present study was relatively small, and larger studies with more animals are needed to confirm and validate the findings. Moreover, the present study only focused on the hippocampus and PFC of mice, and potential changes in other brain regions were not investigated. While mRNA expression data provide compelling evidence of potential BBB damage and increased inflammatory response in our model, it is important to note that mRNA expression does not directly equate to protein function or localization. Future studies are necessary to validate these inflammatory response findings at the protein level or through ELISA assays, while also employing more direct methods to evaluate functional blood–brain barrier permeability (Ben-Zvi et al., 2014; Wood et al., 2021). It is important to note that the findings may not be generalizable to female mice. Given that females are twice as likely as males to receive a diagnosis of AD (Rajan et al., 2021; Lopez-Lee et al., 2024), future studies should include female mice to provide a more comprehensive understanding of the disease across genders.

In summary, our results showed that combination of HFD and STZ/I.P. could induce not only T2DM-like metabolic disorders but also AD-like neuropsychiatric behavior in mice, as indicated by the increased blood glucose levels and serum insulin levels and the impaired cognitive ability in NOR, Y-maze, and MWM tests. Apart from the APP accumulation and tau hyperphosphorylation, the mechanism might be associated with neuron damage and activation of microglia and astrocytes in the hippocampus. Moreover, the imbalanced protein expression of the IRS1/AKT/ERK signaling pathway and TREM1/2, as well as the increased inflammatory factors, may also be involved in AD-like pathological process. These findings might provide new evidence for understanding the pathogenesis of AD-like neuropsychiatric injuries. Future research efforts should focus on further elucidating the underlying mechanisms and identifying potential therapeutic targets for the prevention and treatment of AD.

### Data availability

The original contributions presented in the study are included in the article. Further inquiries can be directed to the corresponding authors.

## Synthesis

Reviewing Editor: Laura Bradfield, University of Technology Sydney Decisions are customarily a result of the Reviewing Editor and the peer reviewers coming together and discussing their recommendations until a consensus is reached. When revisions are invited, a fact-based synthesis statement explaining their decision and outlining what is needed to prepare a revision will be listed below. The following reviewer(s) agreed to reveal their identity: NONE.

Thank you for submitting your work to eNeuro. The reviewers and I have discussed your manuscript and have deemed it to be interesting and, for the most part, suitably executed. The reviewers have made a few specific suggestions that you could use to improve your manuscript but acknowledge that some of the points may be more appropriate to address in future work than current work (e.g. the point about testing at more behavioural time-points raised by reviewer #2). However, there are a number of points made about how the data could be presented more clearly and I would ask that you do your best to address these. If, however, you do not have enough samples to carry out new procedures (for example) perhaps an statement added to the discussion acknowledging the potential limitation raised would suffice. Do feel free to get in contact and ask me any questions you have if you are unsure of how to proceed.

The specific reviews are appended.

Reviewer #1

In this paper "AD-like neuropsychiatric dysfunction in a mice model induced by a combination of high-fat diet and intraperitoneal injection of streptozotocin", the authors provided detailed results of mice models challenged with high-fat diet and streptozotocin, suggesting that combined treatment could induce AD-like behaviors and pathologies, metabolic changes and microglia activations. The study provided some interesting findings and insights into the field. Here are my concerns and comments.

1. I think the authors should first justify why they chose a combined treatment. The study here tried to establish the link between metabolic dysfunction especially insulin resistance and AD. Injection of streptozotocin was enough to induce T2DM. So why included high-fat diet in the experiments? Or did combined treatment provide something more compared to single treatment or other T2DM model?

On the other hand, from the data provided in Figure 1 and 2, high-fat diet was able to induce insulin resistance and cognitive dysfunction, so why further included streptozotocin injection in the experiments? Will this intervention provide further evidence for their hypothesis?

2. In figure 4E, the authors labeled Abeta1-42 at 100kDa, which should not be correct. Could the authors provide full view of the blots?

3. In figure 6, given the blots provided and the N number was just 3, I'm not quite convinced that p-ERK/ERK was significantly decreased in model group. Could the authors provide densitometry results of the blots? And same concerns for Figure 7A and 7D.

4. The investigators used RT-PCR to measure levels of IL-1β, IL-6 and TNF-α. As we know, mRNA levels could only partially represent protein levels. So, I think ELISA would be a better way to measure those cytokines and commercial kits for those cytokines should be available.

5. The manuscript needs more revision to fix grammar problems and some errors.

Reviewer #2

It seems that this study primarily examines AD-like pathological phenotypes within a T2DM model rather than functioning as a primary AD model. This distinction should be clarified in both the abstract and introduction to avoid potential misunderstandings.

Could you please explain the rationale behind using only male mice in this study? Including both genders could provide a more comprehensive understanding.

Given that this is a T2DM model, monitoring weight changes of all mice throughout the experiment would be crucial for a thorough analysis.

To strengthen the study, including additional control groups such as HFD-only and STZ-only groups would be beneficial.

For clarity and transparency, displaying all data points in the quantification plots is recommended.

Including more time points, such as weeks 3, 6, 8, 10, and 12, would help illustrate cognitive function changes throughout the experiments more effectively.

Could you clarify how the FGB was tested at week 8? Was this before or after STZ administration? Additionally, more frequent monitoring of FGB changes would provide valuable insights. How should the increase in FGB at week 8 be interpreted?

Could you specify when the insulin level was tested in Figure 1C?

It is noted that the total distance and average speed of the Mod group differed from the control group at the start, despite the lack of statistical significance. Could you provide an explanation for this observation?

In Figure 3A, it appears that the signal intensity is lower in the Mod group rather than the signal area.

In Figure 3C, the images seem overexposed, making it difficult to discern differences between the control and Mod groups. Additional evidence of neuronal damage or death, such as a TUNEL assay, would strengthen the results.

High-magnification images are needed in Figures 4A and 4C to show more detailed results. Analyzing the colocalization of Ab, p-Tau, and neurons would also be beneficial.

Consider using a more reliable marker for microglial activation, such as CD68, in Figure 5. Including high-magnification images for Figure 5D would enhance the data presentation.

Lastly, assessing BBB integrity in this model using a reliable BBB permeability assessment would add significant value to the study.

Addressing the points above would greatly enhance the robustness and clarity of the study, ensuring clear explanations and justifications for all methods and results presented.

Minor points:

Line 67: "Neurofibrillary tangles" was first shown here.

Line 81: Change to "of T2DM mice."

Line 278: Change "Figure 1B-2C" to "Figure 1B-1C."

Reviewer #2 also notes:

In the methods section, it states that the differences between the two groups were assessed using the Student's t-test. However, the appropriateness of this test depends on the sample size and the sample distribution. The Wilcoxon test should be considered if the sample size is small and the data do not fit a normal distribution.

## Author Response

Reviewer #1 Comment 1: Injection of streptozotocin was enough to induce T2DM. So why included high-fat diet in the experiments? Or did combined treatment provide something more compared to single treatment or other T2DM model? Response: We appreciate your professional comments, which result in a significant improve of the manuscript and throw light to a more scientific design for us, especially more rational group set in our further studies. We appreciate the opportunity to clarify our reasoning behind this choice.

We totally agree with you that streptozotocin (STZ) is an effective inducer of Type 2 Diabetes Mellitus (T2DM), which has also been used widely in the literature(Rakieten et al., 1963). By selectively an directly damaging pancreatic β-cells, intraperitoneal injection of STZ has been demonstrated to result in hyperglycemia and metabolic dysfunction, based on which STZ is used to establish both T2DM and T1DM animal models, with different doses and times(Gu et al., 2023; Vera Castro et al., 2024). However, T2DM is a multifactorial metabolic disease involving a complex interplay of genetics and lifestyle factors, among which diet has been demonstrated to be a vital role in the diabetes incidence and glycemic control(Reed et al., 2000). Consistently, results of our previous studies showed that a high-fat diet could induce not only the non-alcoholic fatty liver disease-like pathological changes, but also hyperglycemia in rats(Gao et al., 2021). Moreover， rats received a ICV or hippocampal injection of STZ could result in both AD-like neuropsychiatric performance and insulin resistance(Fan et al., 2022; Qi et al., 2021). Based on these findings, we tried to investigate the crosstalk between T2DM and AD by combination of high-fat diet and STZ, aiming to mimic the real-world complexity of T2DM, modeling a more clinically relevant scenario that better reflects the metabolic milieu of human T2DM to more accurately mimic the human condition(Furman, 2015). As shown in the manuscript, the increased FBG concentration induced by high-fat diet was further increased after challenge with STZ, together with a significant insulin resistance, which is typical character of T2DM.

In our opinion, the model in this study reflects the pathophysiological process of T2DM more truly and reliably. However, it is regretful that we did not set groups given high-fat diet or STZ alone simultaneously in this study, although we observed the changes of these stimulus alone in our previous studies. Thanks to your professional comments, we will take into account to set groups more rationally when we carry out our further study.

Comment 2: From the data provided in Figure 1 and 2, high-fat diet was able to induce insulin resistance and cogni tive dysfunction, so why further included streptozotocin injection in the experiments? Will this intervention provide further evidence for their hypothesis? Response: Thanks very much for your professional comments.

As we mentioned in answering your comment 1, we establish this model based on our previous studies(Fan et al., 2022; Gao et al., 2023; Gao et al., 2021). By combining HFD and STZ, we aimed to create a more robust model close to the progressive pathological process of human T2DM. As you say, HFD alone can induce obesity, insulin resistance, and metabolic syndrome, which are risk factors for cognitive decline and AD(Liu et al., 2022). The STZ injection serves to exacerbate the metabolic disturbances induced by HFD and also to induce β-cell death, which is a key feature of T2DM and has been linked to cognitive impairment and AD pathology. In this study, our results showed that the combination of HFD and STZ induced not only T2DM-like metabolic disorder, but also cognitive dysfunction. Moreover, the mice presented AD-like neuropathological changes such as increased Aβ and phosphorylated tau in the hippocampus. This dual challenge provides a more accurate representation of the human condition and allows for a more thorough investigation into the complex interplay between metabolism and cognition in AD pathogenesis. In this regard, we would like to say that this intervention could provide further evidence for our hypothesis.

We believe that this approach offers a valuable platform for future research aimed at understanding the molecular mechanisms underlying the link between metabolic disorders and AD, as well as for testing potential therapeutic interventions that target both metabolic and neurodegenerative components of the disease. However, it is regretful that we did not set groups given high-fat diet or STZ alone simultaneously in this study, although we observed the changes of these stimulus alone in our previous studies.

Comment 3: In figure 4E, the authors labeled Abeta1-42 at 100kDa, which should not be correct. Could the authors provide full view of the blots? Response: Thank you for your professional comments. We apologize for the confusion and appreciate the opportunity to clarify this matter.

Upon reviewing our data and the corresponding figure, we agree with you that the label "Aβ" in Figure 4E may indeed be misleading. The band at approximately 100kDa corresponds to the full-length amyloid precursor protein (APP), which is the precursor to Aβ peptides. We regret the oversight in our labeling and the subsequent textual description in the manuscript.

To address this issue, we have taken the following steps:

1. We have reviewed the full Western blot images to ensure accurate labeling and have confirmed that the band at 100kDa corresponds to APP, not Aβ, which is typically observed at a lower molecular weight.

2. We have revised the manuscript to correct the labeling and description of the protein bands in Figure 4E. Specifically, we have updated the figure legend and the text in the Results section to accurately reflect that the 100kDa band represents APP.

3. We have provided a series of images including the Gel Electrophoresis Image, Ponceau S-stained Membrane Image and Corresponding Original Images as follows. This full view clearly shows the ladder and the position of the APP band, which should help to resolve any confusion regarding the identity of the protein.

4. We have also ensured that the APP antibody information is correctly cited in the methodology section, detailing the specific antibody used and its validation for detecting APP. (https://www.ptgcn.com/products/Amyloid-Precursor-Protein,-C-Term inal - Antibody-25524-1-AP.htm#publications) Comment 4: In figure 6, given the blots provided and the N number was just 3, I'm not quite convinced that p-ERK/ERK was significantly decreased in model group. Could the authors provide densitometry results of the blots? And same concerns for Figure 7A and 7D.

Response: Thanks very much for your professional comments. Accordingly, We appreciate your careful scrutiny of our data and your concerns regarding the statistical significance of the findings presented in Figures 6 and 7. In response to your request, we have performed densitometry analysis on the Western blot images to provide a more quantitative assessment of the protein expression changes. To address the concerns about the significance of the p-ERK/ERK ratio and the expression levels of TREM1 and TREM2, we have prepared a Table that includes the densitometry results for these proteins.

Additionally, we have revised our statistical graphs to include individual values, which will provide a clearer representation of the data distribution and variability. Below is a summary of the actions taken:

Revised Statistical Graphs: We have updated all Figures to include individual data points, which will allow readers to visualize the variability within each group and the differences between groups more effectively.

This new table presents the densitometry results for all proteins expression, including the mean, standard deviation, and p-values for the comparisons between the model group and the control group. The table will be included as follow:

Control Model Statistics(t) P-value Mean SEM Mean SEM APP/β-actin Hippocampus 1 0.0783 1.2806 0.02551 -3.408 0.027 PFC 1 0.01209 1.3588 0.03728 -9.154 0.001 p-Tau/Tau Hippocampus 1 0.02238 1.1407 0.04261 -2.295 0.043 PFC 1 0.04751 1.6493 0.01025 -13.359 0.0001 p-IRS1/IRS1 Hippocampus 1 0.06205 1.4273 0.1376 -2.831 0.047 PFC 1 0.05369 1.1616 0.02014 -2.818 0.048 p-AKT/AKT Hippocampus 1 0.01618 0.7981 0.01655 8.721 0.001 PFC 1 0.01921 0.8481 0.03884 3.506 0.025 p-ERK/ERK Hippocampus 1 0.01986 0.9039 0.0136 3.993 0.016 PFC 1 0.02916 0.9098 0.01482 2.758 0.05 TREM1/β-actin Hippocampus 1 0.02575 0.848 0.03642 3.407 0.027 PFC 1 0.01937 0.8924 0.00879 5.058 0.007 TREM2/β-actin Hippocampus 1 0.01934 1.2376 0.02869 -6.866 0.002 PFC 1 0.09412 1.318 0.05335 -2.939 0.042 Comment 5: The investigators used RT-PCR to measure levels of IL-1β, IL-6 and TNF-α. As we know, mRNA levels could only partially represent protein levels. So, I think ELISA would be a better way to measure those cytokines and commercial kits for those cytokines should be available.

Response: We appreciate your insightful comment regarding the measurement of cytokine levels. We totally agree with you that mRNA levels could only partially represent protein levels, and it would be better to measure these parameters using ELISA. Unfortunately, due to the limitations in tissue availability and the scope of our current experimental design, we were unable to add an ELISA experiment in our study.

According to your professional comments, we have listed this as a limitation in the revision. In the discussion section of our manuscript, we acknowledge the limitations of our current study, specifically the reliance on RT-PCR to measure mRNA levels of functional BBB permeability and inflammatory response. We have included the following statement in the discussion section to address this limitation.

The comment results in the changes in line 12-17, page 22 in the revision.

Comment 6: The manuscript needs more revision to fix grammar problems and some errors.

Response: Thanks for your important advice and kind reminder, and we are very sorry for the poor expression. Accordingly, we have checked through all over the manuscript and corrected the awkward sentences and grammar mistakes as far as we can. Moreover, the whole manuscript was revised by Dr. Qiao Jinping, who got his ph.D degree in University of Science and Technology of China, and had a 1 year's post-doctoral experience in College of Medicine, University of Manitoba, Canada, hoping that the new version of our manuscript is easy to understand. If it still needs more modifications, we will improve it as possible as we can, even have it polished by a professional corporation.

The comment results in the changes mainly in the methods and materials, together with lots of minor modifications scattered in the revision.

Reviewer #2 Comment 1: It seems that this study primarily examines AD-like pathological phenotypes within a T2DM model rather than functioning as a primary AD model. This distinction should be clarified in both the abstract and introduction to avoid potential misunderstandings.

Response: We appreciate your professional comments and kind suggestion. Accordingly, we have revised and improved the description in the revision. Indeed, as you mentioned, the aim of this study was to investigate the AD-like pathological phenotypes within the context of a T2DM model.

Revision for the abstract:Although AD and T2DM are distinct pathologies, our results suggested that combination of HFD and STZ/I.P. a widely used T2DM modeling method, could successfully induce AD-like behavioral impairments and neuropathological injuries in mice, the mechanism might be involved with neuroinflammation and its associated dysfunction of IRS1/AKT/ERK signaling pathway. Our findings further support the potential overlap between T2DM and AD pathophysiology, providing insight into the mechanisms underlying the comorbidity of these diseases.

And for the introduction: Focusing on the intersection of T2DM and AD, we aim to elucidate the mechanisms by which metabolic dysfunction may contribute to neurodegenerative changes.

We hope that these revisions will provide the necessary clarity and help readers to correctly interpret the purpose and findings of our study. We thank you for your valuable feedback, which will undoubtedly enhance the manuscript's overall quality and readability.

The comment results in the changes in line20-25, page 1 and line16-17, page 5 in the revision.

Comment 2: Could you please explain the rationale behind using only male mice in this study? Including both genders could provide a more comprehensive understanding.

Response: We appreciate your insightful comments. We totally agree with you that including both genders could provide a more comprehensive understanding. However, we enrolled only male mice in the present study. This design was based on several factors that are commonly considered in preclinical research, among which the standardization is the most important one. Although according to the Alzheimer's Disease Report of China, the prevalence and mortality rate of women are higher than that of men, which the prevalence rate of women is about 1.8 times that of men and the death rate for women is more than twice that of men. However, mainly due to the consideration of standardization, we want to exclude the potential confounding effects of estrous cycles and hormonal fluctuations in females, which might complicate the interpretation of the results. Although it has also been reported that the estrous cycles would be synchronized in a closed colony in rodents, most studies focusing on metabolic diseases and neuropsychiatric diseases use only male animals(Yesiltepe et al., 2024; Zou et al., 2024). Additionally, it has been reported that male mice tend to be less variable in terms of body weight and metabolic parameters compared to females(Braga Tibaes et al., 2024; Mauvais-Jarvis, 2024).

Comment 3: Given that this is a T2DM model, monitoring weight changes of all mice throughout the experiment would be crucial for a thorough analysis.

Response: We thank you for highlighting the importance of monitoring weight changes in the context of a T2DM model. You are absolutely right that tracking weight changes throughout the experiment is crucial for a thorough analysis, as weight gain or loss can be a significant indicator of metabolic health and disease progression in diabetic conditions.

In response to your suggestion, we have included the weight changes of all mice throughout the experiment in Figure 1E. This figure now provides a visual representation of the weight dynamics over time for both the control and model groups. We believe that this update will address your concerns contribute to a more robust interpretation of the study's findings.

The comment results in the changes in line1-4, page 13 in the revision.

Comment 4: To strengthen the study, including additional control groups such as HFD-only and STZ-only groups would be beneficial.

Response: We acknowledge and appreciate your suggestion to include additional control groups, such as the HFD-only and STZ-only groups, in our study. Your recommendation to include these groups would indeed enhance the study by providing a more comprehensive understanding of the individual effects of each intervention, as well as how they interact to produce the outcomes we have observed.

We totally agree with you that streptozotocin (STZ) is an effective inducer of Type 2 Diabetes Mellitus (T2DM), which has also been used widely in the literature(Rakieten et al., 1963). By selectively an directly damaging pancreatic β-cells, intraperitoneal injection of STZ has been demonstrated to result in hyperglycemia and metabolic dysfunction, based on which STZ is used to establish both T2DM and T1DM animal models, with different doses and times(Gu et al., 2023; Vera Castro et al., 2024). However, T2DM is a multifactorial metabolic disease involving a complex interplay of genetics and lifestyle factors, among which diet has been demonstrated to be a vital role in the diabetes incidence and glycemic control(Reed et al., 2000). Consistently, results of our previous studies showed that a high-fat diet could induce not only the non-alcoholic fatty liver disease-like pathological changes, but also hyperglycemia in rats(Gao et al., 2021). Moreover， rats received a ICV or hippocampal injection of STZ could result in both AD-like neuropsychiatric performance and insulin resistance(Fan et al., 2022; Qi et al., 2021). Based on these findings, we tried to investigate the crosstalk between T2DM and AD by combination of high-fat diet and STZ, aiming to mimic the Real-World Complexity of T2DM, modeling a more clinically relevant scenario that better reflects the metabolic milieu of human T2DM to more accurately mimic the human condition(Furman, 2015). As shown in the manuscript, the increased FBG concentration induced by high-fat diet was further increased after challenge with STZ, together with a significant insulin resistance, which is typical character of T2DM.

In our opinion, the model in this study reflects the pathophysiological process of T2DM more truly and reliably. However, it is regretful that we did not set groups given high-fat diet or STZ alone simultaneously in this study, although we observed the changes of these stimulus alone in our previous studies. Thanks to your professional comments, we will take into account to set groups more rationally when we carry out our further study.

Comment 5: For clarity and transparency, displaying all data points in the quantification plots is recommended.

Response: We appreciate your emphasis on clarity and transparency in data presentation. In response to your recommendation, we have indeed included all data points in the quantification plots. Each plot now clearly displays individual data points for both the control and model groups, along with standard error of the mean (SEM).

This approach allows readers to visualize the distribution of the data and the variability within each group, providing a more accurate representation of the results. Additionally, it helps to ensure that the conclusions drawn from the data are based on a full understanding of the underlying data points.

In the methods section of our manuscript, we have also described this approach to data presentation, explaining that all data points were included in the plots to maintain transparency and facilitate a comprehensive analysis of the results. We believe that these changes will enhance the readability of the manuscript and provide a clearer picture of the data for our audience. We thank you for your suggestion and for helping us to improve the quality of our presentation.

The comment results in the changes in line12-14, page 12 in the revision.

Comment 6: Including more time points, such as weeks 3, 6, 8, 10, and 12, would help illustrate cognitive function changes throughout the experiments more effectively.

Response: We appreciate your professional comments and valuable suggestions. We totally agree with you that it would be better to measure the parameters with more time point, which would provide more information to observe the dynamical changes during the process of T2DM. However, we just selected 2 time point in this study, one was before administration of STZ, and the other was the end of the total experiment. It is regretful that we are unable to retroactively include additional time points in the current study, as the experiments have already been completed.

We fully recognize this limitation and appreciate the your professional comments, which will reminder us to improve our further experimental designs with more rational consideration.

Comment 7: Could you clarify how the FGB was tested at week 8? Was this before or after STZ administration? Additionally, more frequent monitoring of FGB changes would provide valuable insights. How should the increase in FGB at week 8 be interpreted? Response: Thank you for your professional comments regarding the timing of the FGB test at week 8 and the interpretation of the observed changes. To clarify, the FGB measurements were conducted before the administration of STZ, ensuring that the values reflect the metabolic status of the mice following the HFD alone.

For measuring the FBG in week 8, the blood glucose levels were measured using a Roche blood glucose meter (Accu-Chek line, Roche Diagnostics, Indianapolis, IN, USA) in combination with Roche Accu-Chek test strips after an overnight fast. The mice were gently pricked with a lancet on the tail tip to obtain a small blood sample. A drop of blood was applied to the test strip, and the glucose level was read on the glucose meter according to the manufacturer's instructions.

We totally agree with you that more frequent monitoring of FGB changes would provide valuable insights. One of the reasons for the inadequate time point was that the mice was continued to raise after detection of FBG and behavioral tasks in week 8. Although the blood volume is little, we considered that too many times of blood collection would bring harm and adverse effect to the animals. We are unable to add more frequent assessments of FGB in this study now. However, according to your professional comments, we will adopt more time point to observe the dynamical changes more rationally in our further study, maybe it could be fulfilled to get samples from different patches of mice modeling simultaneously in the same way.

Regarding the interpretation of the increase in FGB at week 8, it is important to note that HFD itself can lead to an increase in blood glucose levels due to the development of insulin resistance and the subsequent impairment of glucose metabolism. It has been reported that both rats and mice, breed with HFD, 8 weeks or longer time, could induce a significant metabolic disorders including hyperglycemia, hyperlipidemia, and liver dysfunction(Kambham et al., 2001; Winzell and Ahrén, 2004).

Comment 8: Could you specify when the insulin level was tested in Figure 1C? Response: Thank you very much for your professional comments. In response to your inquiry regarding the timing of the insulin level test depicted in Figure 1C, we can confirm that the serum insulin concentrations were measured after the mice were sacrificed. Specifically, each mice was fasted overnight and deep anesthetized, and then the blood was collected and the serum was isolated by centrifugation at 3000 rpm for 15 min. The serum insulin levels were then determined using a commercial insulin ELISA kit following the procedure outlined in the manuscript's methodology section.

For clarity, here is the relevant detail from the manuscript: "Serum insulin concentrations were measured in fasting mice (fed the night before and fasted for 12-16 hours prior to sacrifice) using a commercially available insulin ELISA kit (Wuhan ColorfulGene Biological Technology Co., LTD, Wuhan, China). Blood was collected by cardiac puncture immediately after sacrifice, and serum was separated and stored at -80{degree sign}C until analysis. All measurements were performed in accordance with the manufacturer's instructions.

The comment results in in the changes in line12-19, page 9 in the Measurement of serum samples.

Comment 9: It is noted that the total distance and average speed of the Mod group differed from the control group at the start, despite the lack of statistical significance. Could you provide an explanation for this observation? Response: Thank you for your astute observation regarding the differences in total distance and average speed between the Mod group and the control group at the start of the experiment, despite these differences not reaching statistical significance.

This observation may be attributed to Random Chance: In some cases, the differences observed may simply be due to random chance, particularly if the sample size is small. With a larger sample size, these initial differences may not be apparent due to the averaging effect.

To further clarify the impact of initial differences on our study's outcomes, we have indeed included a repeated measures design in our experimental protocol. This approach allows us to assess whether the observed differences between the Mod group and the control group are persistent or change over time, providing a deeper understanding of the stability of these differences and their relevance to the experimental results. Results of the repeated measures data has shown that: Any initial differences in total distance and average speed between the groups did not significantly affect the subsequent comparisons. The differences, whether they were present initially or emerged later, were consistent across multiple testing sessions, suggesting a stable effect related to the experimental conditions. The repeated measures have helped us to confirm that the initial observations did not confound or bias our final conclusions. Any initial variations were accounted for in our statistical analyses, ensuring that the comparisons made between the groups later in the study are valid and robust. Additionally, as shown in the y-axis of the figure, the difference of the specific values of the measured parameters was indeed very little.

By incorporating this analysis, we aim to reinforce the integrity of our experimental approach and to provide a clear rationale for why initial differences did not affect our final interpretations. We hope this will satisfy the reviewer's concerns and enhance the manuscript's clarity and scientific rigor.

Comment 10: In Figure 3A, it appears that the signal intensity is lower in the Mod group rather than the signal area.

Response: Thank you for pointing out the specific detail in Figure 3A. You are correct in observing that the figure illustrates a difference in signal intensity rather than signal area for MAP-2 expression in the hippocampal region between the Mod group and the control group.

To address this and ensure clarity in our manuscript, we will make the following corrections and clarifications: In the text where Figure 3A is discussed, we will ensure that the language used describes the decrease in fluorescence intensity rather than the signal area. "the Mod group exhibited significantly lower fluorescence intensity for MAP-2 in the hippocampal region, suggesting a reduction in the expression of this neuronal marker compared to the control group." The comment results in the changes in line24-25, page 13 in the revision.

We appreciate your attention to detail, and we believe these revisions will enhance the manuscript's clarity and ensure that the findings are reported with the highest level of precision.

Comment 11: In Figure 3C, the images seem overexposed, making it difficult to discern differences between the control and Mod groups. Additional evidence of neuronal damage or death, such as a TUNEL assay, would strengthen the results.

Response: We acknowledge your concern regarding the overexposure of images in Figure 3C, which may indeed obscure the visualization of differences between the control and Mod groups. Clarity in figures is crucial for the interpretation of results.

In response to your comments, we have taken the following actions: Replacement of Images: We have replaced the overexposed images with new ones that provide a clearer representation of the hippocampal region. The new images have been carefully adjusted to ensure that the details are visible, allowing for a more accurate comparison between the control and Mod groups.

TUNEL Assay Consideration: We appreciate your suggestion for additional evidence to support our findings. While we currently do not have TUNEL assay data due to the unavailability of tissue samples, we agree that such evidence could strengthen our results. We are planning to include a TUNEL assay in our future studies to provide additional evidence of neuronal damage or death.

Comment 12: High-magnification images are needed in Figures 4A and 4C to show more detailed results. Analyzing the colocalization of Ab, p-Tau, and neurons would also be beneficial.

Response:We appreciate your suggestion for including high-magnification images to provide a more detailed view of the results presented in Figures 4A and 4C. The additional request for analyzing the colocalization of Aβ, p-Tau, and neuronal markers is also a valuable contribution that can enhance the understanding of the interactions between these proteins in the context of neuronal health.

In compliance with the reviewer's suggestions, we have incorporated high-magnification images for Figures 4A and 4C. These images now provide a more granular view of the staining patterns, allowing for a more nuanced interpretation of the data.

Furthermore, we have conducted a colocalization of APP and neuronal markers. The results of this analysis are presented in Figure. 4E, which clearly illustrates the degree of overlap between these proteins within the neuronal cells. This colocalization study adds a layer of depth to our understanding of the potential interactions between these molecules in the context of neurodegenerative diseases.

We hope that these enhancements will not only improve the manuscript's quality but also facilitate a better understanding of the complex biological processes under investigation.

The comment results in the changes in line15-16, page 14 in the revision.

Comment 13: Consider using a more reliable marker for microglial activation, such as CD68, in Figure 5. Including high-magnification images for Figure 5D would enhance the data presentation.

Response: We appreciate the reviewer's suggestion to use CD68 as a marker for microglial activation. We agree that CD68 is a robust and widely accepted marker for this purpose. In response, we have incorporated co-staining for microglial cells with CD68, which is a widely accepted marker of activated microglia. The revised Figure 5D now includes this co-staining, providing a more robust demonstration of microglial activation in the hippocampus of the Mod mice compared to the Con mice. This addition enhances the credibility of the findings and should satisfy the reviewer's concerns regarding the specificity of microglial activation markers used in the study.

Regarding the high-magnification images for Figure 5E, we acknowledge the importance of these images for a more detailed analysis of astrocytes morphology and distribution. We are very sorry that we have not provide these images in the initial submission, and we are now in a position to supply the requested high-magnification images. These images will be included in the revised manuscript to provide a clearer representation of the astrocytes activation in the regions of interest.

The comment results in the changes in line 4-5, page 15 in the revision.

Comment 14: Lastly, assessing BBB integrity in this model using a reliable BBB permeability assessment would add significant value to the study.

Response:We are grateful for your suggestion regarding the assessment of blood-brain barrier (BBB) integrity. You are correct that evaluating BBB permeability is crucial for understanding the pathophysiological processes in neurodegenerative diseases and other conditions affecting the central nervous system.

In response to your recommendation, we have conducted an analysis of mRNA expression levels for several key tight junction proteins that are critical for maintaining BBB integrity. These proteins include occludin (ocln), zonula occludens-1 (zo-1), and claudin-5 (clnds).

The following is the revised part of our manuscript: "the mRNA expression levels of ocln, zo-1, and clnds in the hippocampus and PFC of Mod mice were all remarkably increased as compared with the Con ones, which suggesting indicating potential BBB damage induced by the combination of HFD and STZ/I.P. in mice." In discussion, we also further elaborate on the limitations and the need for future research: "While mRNA expression data provide compelling evidence of potential BBB damage and increased inflammatory response in our model, it is important to note that mRNA expression does not directly equate to protein function or localization. Therefore, our findings should be interpreted with caution, and future studies are warranted to confirm these observations at the protein level or ELISA assays to assess functional BBB permeability and inflammatory response." These findings suggest that the BBB may be compromised in our model, which could have implications for the pathogenesis of the disease and the effectiveness of therapeutic strategies. The inclusion of these data in Supplementary Figure 1 provides additional evidence of the physiological changes associated with the disease state in our model. We are confident that this will address your concerns and contribute to a deeper understanding of the disease mechanisms under investigation.

The comment results in the changes in line5-8, page 14 and line12-17, page 22 in the revision.

Comment 15: Minor points:

Line 67: "Neurofibrillary tangles" was first shown here.

Line 81: Change to "of T2DM mice." Line 278: Change "Figure 1B-2C" to "Figure 1B-1C." Response: Thanks for your professional comments, and we would like to express our sincere gratitude for your meticulous review of our manuscript. Accordingly, we have revised these points and checked through all over the manuscript to correct when necessary.

The comments resulted in the changes in line16, page 3; line23, page 3; line24, page 12; and other changes scattering in the revision.

Comment 16: In the methods section, it states that the differences between the two groups were assessed using the Student's t-test. However, the appropriateness of this test depends on the sample size and the sample distribution. The Wilcoxon test should be considered if the sample size is small and the data do not fit a normal distribution.

Response: Thank you for your valuable feedback. We appreciate your attention to the statistical methods used in our study.

In response to your comment regarding the use of the Student's t-test, we have re-evaluated our methodology. We acknowledge that the appropriateness of the Student's t-test indeed depends on the sample size and the distribution of the data. In light of your suggestion, we have conducted the following additional analyses:

Sample Size and Distribution Check: We have re-assessed the sample size and distribution of our data. Although our sample size is within an acceptable range for the Student's t-test, we agree that it is important to consider alternative tests to ensure robustness.

Wilcoxon Test: To address this, we have now also performed the Wilcoxon rank-sum test (Mann-Whitney U test) on our data. The results of the Wilcoxon test are consistent with those obtained from the Student's t-test, indicating that our conclusions remain valid regardless of the test used.

The revised methods section now includes the following statement:"Although the differences between the two groups were initially assessed using the Student's t-test, we also conducted the Wilcoxon rank-sum test to account for potential deviations from normality and small sample size.

The comment results in the changes in line 5-9, page 2 in the revision.

Reference Braga Tibaes, J.R., Barreto Silva, M.I., Wollin, B., Vine, D., Tsai, S., and Richard, C. (2024). Sex differences in systemic inflammation and immune function in diet-induced obesity rodent models: A systematic review. Obes Rev 25, e13665.

Fan, M., Liu, S., Sun, H.M., Ma, M.D., Gao, Y.J., Qi, C.C., Xia, Q.R., and Ge, J.F. (2022). Bilateral intracerebroventricular injection of streptozotocin induces AD-like behavioral impairments and neuropathological features in mice: Involved with the fundamental role of neuroinflammation. Biomed Pharmacother 153, 113375.

Furman, B.L. (2015). Streptozotocin-Induced Diabetic Models in Mice and Rats. Curr Protoc Pharmacol 70, 5.47.41-45.47.20.

Gao, X., Wei, Y., Sun, H., Hao, S., Ma, M., Sun, H., Zang, D., Qi, C., and Ge, J. (2023). Role of Bmal1 in Type 2 Diabetes Mellitus-Related Glycolipid Metabolic Disorder and Neuropsychiatric Injury: Involved in the Regulation of Synaptic Plasticity and Circadian Rhythms. Mol Neurobiol 60, 4595-4617.

Gao, X.R., Chen, Z., Fang, K., Xu, J.X., and Ge, J.F. (2021). Protective effect of quercetin against the metabolic dysfunction of glucose and lipids and its associated learning and memory impairments in NAFLD rats. Lipids Health Dis 20, 164.

Gu, L., Cui, X., Lin, X., Yang, J., Wei, R., Hong, T., and Yang, K. (2023). γ-aminobutyric acid modulates α-cell hyperplasia but not β-cell regeneration induced by glucagon receptor antagonism in type 1 diabetic mice. Acta Diabetol 60, 19-28.

Kambham, N., Markowitz, G.S., Valeri, A.M., Lin, J., and D'Agati, V.D. (2001). Obesity-related glomerulopathy: an emerging epidemic. Kidney Int 59, 1498-1509.

Liu, P., Wang, Z.H., Kang, S.S., Liu, X., Xia, Y., Chan, C.B., and Ye, K. (2022). High-fat diet-induced diabetes couples to Alzheimer's disease through inflammation-activated C/EBPβ/AEP pathway. Mol Psychiatry 27, 3396-3409.

Mauvais-Jarvis, F. (2024). Sex differences in energy metabolism: natural selection, mechanisms and consequences. Nat Rev Nephrol 20, 56-69.

Qi, C.C., Chen, X.X., Gao, X.R., Xu, J.X., Liu, S., and Ge, J.F. (2021). Impaired Learning and Memory Ability Induced by a Bilaterally Hippocampal Injection of Streptozotocin in Mice: Involved With the Adaptive Changes of Synaptic Plasticity. Front Aging Neurosci 13, 633495.

Rakieten, N., Rakieten, M.L., and Nadkarni, M.R. (1963). Studies on the diabetogenic action of streptozotocin (NSC-37917). Cancer Chemother Rep 29, 91-98.

Reed, M.J., Meszaros, K., Entes, L.J., Claypool, M.D., Pinkett, J.G., Gadbois, T.M., and Reaven, G.M. (2000). A new rat model of type 2 diabetes: the fat-fed, streptozotocin-treated rat. Metabolism 49, 1390-1394.

Vera Castro, M.F., Assmann, C.E., Reichert, K.P., Coppetti, P.M., Stefanello, N., da Silva, A.D., Mostardeiro, V.B., de Jesus, L.B., da Silveira, M.V., Schirmann, A.A., et al. (2024). Vitamin D3 mitigates type 2 diabetes induced by a high carbohydrate-high fat diet in rats: Role of the purinergic system. J Nutr Biochem 127, 109602.

Winzell, M.S., and Ahrén, B. (2004). The high-fat diet-fed mouse: a model for studying mechanisms and treatment of impaired glucose tolerance and type 2 diabetes. Diabetes 53 Suppl 3, S215-219.

Yesiltepe, M., Yin, T., Tambini, M., Bao, H., Pan, M., d'Abramo, C., Giliberto, L., Han, X., and Luciano, D. (2024). Analysis of early effects of human APOE isoforms on Alzheimer's disease and type III hyperlipoproteinemia pathways using knock-in rat models with humanized APP and APOE. Cell Commun Signal 22, 458.

Zou, J., Song, Q., Shaw, P.C., Wu, Y., Zuo, Z., and Yu, R. (2024). Tangerine Peel-Derived Exosome-Like Nanovesicles Alleviate Hepatic Steatosis Induced by Type 2 Diabetes: Evidenced by Regulating Lipid Metabolism and Intestinal Microflora. Int J Nanomedicine 19, 10023-10043.
